# Whole-Brain Mapping in Adult Zebrafish and Identification of the Functional Brain Network Underlying the Novel Tank Test

**DOI:** 10.1523/ENEURO.0382-24.2025

**Published:** 2025-03-20

**Authors:** Neha Rajput, Kush Parikh, Ada Squires, Kailyn K. Fields, Matheu Wong, Dea Kanani, Justin W. Kenney

**Affiliations:** Department of Biological Sciences, Wayne State University, Detroit, Michigan 48202

**Keywords:** brain mapping, exploratory behavior, functional brain network, functional connectome, light-sheet microscopy, image registration

## Abstract

Zebrafish have gained prominence as a model organism in neuroscience over the past several decades, generating key insight into the development and functioning of the vertebrate brain. However, techniques for whole-brain mapping in adult stage zebrafish are lacking. Here, we describe a pipeline built using open-source tools for whole-brain activity mapping in adult zebrafish. Our pipeline combines advances in histology, microscopy, and machine learning to capture *c-fos* activity across the entirety of the brain. Following tissue clearing, whole-brain images are captured using light-sheet microscopy and registered to the recently created adult zebrafish brain atlas (AZBA) for automated segmentation. By way of example, we used our pipeline to measure brain activity after zebrafish were subject to the novel tank test, one of the most widely used behaviors in adult zebrafish. *c-fos* levels peaked 15 min following behavior and several regions, including those containing serotoninergic and dopaminergic neurons, were active during exploration. Finally, we generated a novel tank test functional brain network. This revealed that several regions of the subpallium form a cohesive subnetwork during exploration. Functional interconnections between the subpallium and other regions appear to be mediated primarily by ventral nucleus of the ventral telencephalon (Vv), the olfactory bulb, and the anterior part of the parvocellular preoptic nucleus (PPa). Taken together, our pipeline enables whole-brain activity mapping in adult zebrafish while providing insight into neural basis for the novel tank test.

## Graphical abstract

**Figure:**
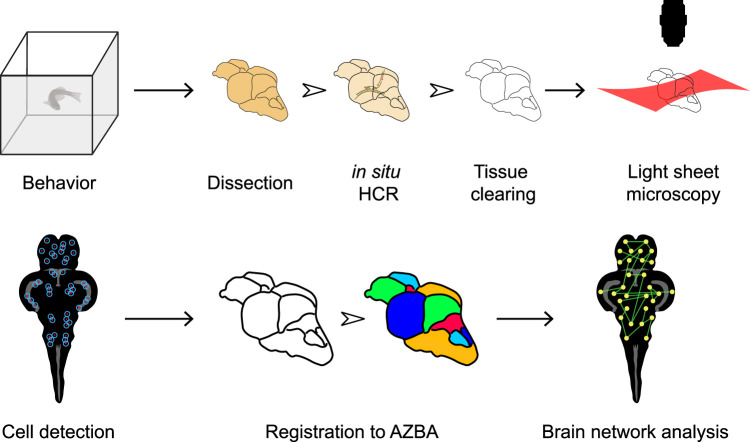
Visual Abstract

## Significance Statement

Zebrafish have grown in popularity as a model organism over the past several decades due to their low cost, ease of genetic manipulation, and similarity to other vertebrates like humans and rodents. However, to date, tools for whole-brain mapping in adult stage animals has been lacking. Here, we present an open-source pipeline for whole-brain mapping in adult zebrafish. We demonstrate the use of our pipeline by generating a functional brain network for one of the most widely used behavioral assays in adult zebrafish, the novel tank test. We found that exploration of a novel tank engages the olfactory bulb and a network of subpallial regions that correspond to the mammalian subpallial amygdala and basal ganglia.

## Introduction

A fundamental goal of neuroscience is to understand how patterns of brain activity give rise to behavior. Identifying general principles of brain function is facilitated by cross-species comparisons. Over the past several decades, zebrafish have contributed to our understanding of the brain, a trend that promises to continue due to their low cost, ease of genetic manipulation, and sophisticated behavioral repertoire (Grunwald et al., 1988; Brockerhoff et al., 1995; Kenney, 2020; Loring et al., 2020; Burgess and Burton, 2023; Gerlai, 2023). Although several methods have been developed for whole-brain activity mapping in larval zebrafish (Ahrens et al., 2012; Portugues et al., 2014; Randlett et al., 2015; Shainer et al., 2023), equivalent approaches have yet to be developed for adult stage animals.

Adult and larval zebrafish each have distinct advantages and disadvantages in the study of brain–behavior relationships. Whereas larval animals are amenable to high-throughput work and live brain imaging due to their small size and transparency, adults have the advantage of mature neuroanatomy and more extensive behavioral repertoire. This adult behavioral repertoire includes a variety of social behaviors (Gerlai, 2014; Jones and Norton, 2015; Kareklas et al., 2023); short- and long-term associative, nonassociative, and spatial memories (Gerlai, 2020; Kenney, 2020); and different types of exploratory behaviors (Cachat et al., 2010; Toms and Echevarria, 2014; Rajput et al., 2022). Thus, to fully realize the utility of zebrafish as a model organism in neuroscience, methods for whole-brain mapping are also required for adult zebrafish.

Whole-brain activity mapping can yield unexpected insights into brain function that may be lost using more targeted methods. Measuring neural activity across the entire brain also facilitates the use of powerful analytic tools, like network analysis that captures complex interactions that are a hallmark of brain function and improves predictions of brain–behavior relationships (Bullmore and Sporns, 2009; Wheeler et al., 2013; Vetere et al., 2017). However, mapping whole-brain activity presents several technical challenges. One roadblock is that the brain of adult animals is not transparent and thus requires the use of tissue clearing (Richardson et al., 2021). Imaging intact organs presents another technical hurdle due to the increased volume, a challenge met by the recent development of light-sheet microscopy (Hillman et al., 2019). Finally, whole-brain mapping results in large amounts of data that cannot be analyzed via traditional approaches like manual cell counting and segmentation. This last challenge was met by combining advances in machine learning to automate cell detection (Tyson et al., 2021) and image registration (Gholipour et al., 2007) to the digital adult zebrafish brain atlas (AZBA; Kenney et al., 2021). Here, we describe our pipeline of open-source tools to enable whole-brain mapping in adult zebrafish and demonstrate an application by identifying a functional brain network that underlies the novel tank test.

## Materials and Methods

### Subjects and behavior

#### Zebrafish

Subjects were 8–10-month-old zebrafish of the TU strain from both sexes. Fish were bred and raised at Wayne State University and within two generations of animals obtained from the Zebrafish International Resource Center (ZIRC, catalog ID: ZL84) at the University of Oregon. Fish were maintained in high-density racks under standard conditions: water temperature of 27.5 ± 0.5°C, salinity of 500 ± 10 µS, and pH of 7.4 ± 0.2. Lighting followed a 14:10 h light/dark cycle, with lights on at 8:00 A.M. Fish were fed twice daily with dry feed (Gemma 300, Skretting) in the morning and brine shrimp (*Artemia salina*, Brine Shrimp Direct) in the afternoon.

Sex determination was based on secondary sex characteristics such as shape, color, and the presence of pectoral fin tubercles (McMillan et al., 2015). Confirmation was conducted postexperimentation by killing the animals and observing the presence or absence of eggs. All experimental procedures were conducted under the ethical approval of the Wayne State University Institutional Animal Care and Use Committee (Protocol ID: 21-02-3238).

#### Behavioral stimuli and tissue collection

The novel tank test was used as the behavioral stimulus, using tanks that were distinct from housing tanks. Behavioral tanks were open top five-sided (15 × 15 × 15 cm) and made from frosted acrylic (TAP Plastics). Each tank was filled to a height of 12 cm with 2.5 L of fish facility water and housed within a white corrugated plastic enclosure to minimize external disturbances and diffuse light.

One week before the novel tank test, animals were housed in 2 L tanks divided into two chambers with transparent dividers. Male and female pairs were kept in each chamber to enable identification of individuals without social isolation or tagging. A day prior to the experiment, animals were acclimatized to the behavior room for 1 h before being placed back on the housing racks. On the day of the experiment, animals were removed from the housing rack and allowed to acclimate in the behavioral room for 1 h. After acclimation, animals were individually transferred to a novel tank and allowed to explore the tank for 6 min. After 6 min, fish were removed and placed back in their home tank for a designated periods of time (5, 15, 30, 60, or 120 min) prior to killing. Water was replaced between animals. A subset of animals was killed 1 h after acclimation to the room (home tank control) and another set of animals were killed immediately after removal from the housing racks (rack control).

Animals were killed by immersion in ice-cold water for 5 min to induce anesthesia and then decapitated using a sharp blade. We found no difference in *c-fos* cell counts whether fish were immersed in cold water for 1 or 5 min (unpublished observations). Heads were then washed in ice-cold phosphate-buffered saline (PBS) for 60 s to allow for blood drainage and then fixed in 4% paraformaldehyde in PBS at 4°C overnight. Brains were then dissected in ice-cold PBS and subject to iDISCO and in situ HCR.

### Histology

#### Tissue pretreatment

We adapted the iDISCO protocol (Renier et al., 2016) for zebrafish brain tissue staining. Following dissection, brain samples were washed for 30 min, three times, in PBS at room temperature. This was followed by dehydration using a methanol concentration gradient (20, 40, 60, 80, and 100%) for 30 min each at room temperature. Samples were further washed in 100% methanol, chilled on ice, and then incubated in chilled 5% hydrogen peroxide in methanol overnight at 4°C. The next day, the samples were rehydrated through a reverse methanol series (80, 60, 40, and 20%) at room temperature, followed by a 1 h PBS wash, two 1 h PBS-T washes (1× PBS, 0.1% Tween 20), and a 3 h PBS-T wash. Samples were then equilibrated overnight in 5× SSCT (sodium chloride sodium citrate/0.1% Tween-20) buffer.

#### In situ HCR and tissue clearing

We modified the original HCR method described by Choi et al. (2018) and informed by the work of Kumar et al. (2021). These modifications include the initial acetylation step to reduce background and reduced volumes and decreased volume of HCR amplification buffer to reduce waste and increased concentration of hairpins to increase the signal strength. After dissection, samples were acetylated in 0.25% v/v acetic anhydride solution in ultrapure water for 30 min. Samples were then washed in ultrapure water three times for 5 min and then equilibrated in probe hybridization buffer (30% formamide, 5× SSC, 9 mM citric acid, 0.1% Tween-20, 50 µg/ml heparin, 1× Denhardt's solution, 10% dextran sulfate) for 15 min at room temperature. Samples were then incubated in probe hybridization buffer for 1 h at 37°C with shaking and then incubated with 1 µM of *c-fos* probes (Molecular Instruments) in hybridization buffer at 37°C with shaking for 48–60 h. Samples were then washed with probe wash buffer (30% formamide, 5× SSCT, 9 mM citric acid, 50 µg/ml heparin) three times at 37°C and then twice with 5× SSCT for 1 h each with shaking. The tissue was then equilibrated in amplification buffer (5× SSC, 0.1% Tween-20, 10% dextran sulfate) at room temperature for 1 h with shaking. Alexa647 labeled hairpins (B1; Molecular Instruments) were prepared by heating to 95°C for 90 s prior to cooling at room temperature in the dark. We diluted 7.5 pmol of each hairpin into 125 µl of amplification buffer for each sample. Samples were incubated for 48–60 h in the dark at room temperature. Finally, tissue was washed in 5× SSCT, five times for 1 h each before being washed overnight in 5× SSCT.

Following the last 5× SSCT wash, samples were dehydrated in a series of methanol–water mixtures (20, 40, 60, 80, 100%) at room temperature for 1 h each and then left in 100% methanol overnight. The next day, samples were incubated at room temperature in a mixture of 66% dichloromethane and 33% methanol for 3 h followed by two 15 min washes in dichloromethane. After removing the dichloromethane, samples were incubated and stored in dibenzyl ether at room temperature for at least 24 h until imaging.

#### Brain imaging and processing

Cleared samples were imaged on a LaVision BioTec UltraMicroscope II (Miltenyi Biotec) using Imspector software for image acquisition. The microscope setup included a 4.2 megapixel sCMOS camera and a 2× objective lens with a dipping cap with spherical aberration correction. Images were taken at a magnification of 6.4×. Samples were mounted on the sample holder using an ultraviolet cured resin (NOA 61, Norland Products) with a refractive index (1.56) that matched DBE (Extended Data Fig. 2-1). Imaging was conducted from the right laser with a 4 μm step size using dynamic horizontal focus. Both 480 nm autofluorescence and 640 nm signal channels were used. The imaging settings used were the following: 90 and 20% laser power for the 640 and 480 nm lasers, respectively, 200 ms exposure time, 50% sheet width, and a sheet numerical aperture of 0.156. Acquired images were stitched using TeraStitcher (Bria and Iannello, 2012).

### Computational analysis

#### Automated cell detection

We used CellFinder (Tyson et al., 2021) for the automated detection and quantification of *c-fos*-positive cells. It comprises two steps: cell candidate detection and cell classification. The initial step of cell detection identifies cell-like objects in the image. We optimized parameters to capture as many cell-like objects in our images as possible. Running from the Linux terminal, we used the following command for cell detection:

cellfinder -s path/to/folder/signal/channel/c-fos -b /path/to/folder/background/channel/AF -o path/to/output1 -v 3.990 0.943 0.943 –orientation sal –no-register –no-classification –soma-diameter 5 –threshold 3 –ball-xy-size 2 –ball-z-size 7 –ball-overlap-fraction 0.77 –log-sigma-size 0.1 –save-csv –batch-size 64 –epochs 100

After detecting cell candidates, a customized Python script was used to remove cell candidates that were within 9 μm of one another. This last step was done because the cell detection algorithm tended to double count cells; altering parameters to remove the double counting resulted in unacceptable loss of cell detection in other parts of the brain. We chose the 9 μm empirically by exploring several distances, finding that this distance resulted in the removal of overlapping candidates without unduly affecting the detection of distinct cells in close proximity.

Napari was used for visualization and labeling. We manually annotated 10,597 cells and 7,303 noncells across five brains for training the artificial neural network. CellFinder was trained using the following command:

Cellfinder_train -y path/to/brain1_labels.yml path/to/brain2_labels.yml path/to/brain3_labels.yml path/to/brain4_labels.yml path/to/brain5_labels.yml -o /trained_network –batch-size 64 –epochs 100 –no-save-checkpoints –save-progress

The trained network achieved 96.1% accuracy. Finally, the trained network was applied to all the experimental brains to classify the detected cell candidates into cells and noncells. This was achieved by the following command:

cellfinder -s /path/to/folder/signal/channel/c-fos/ -b /path/to/folder/background/channel/AF/ -o path/to/output -v 3.990 0.943 0.943 –orientation sal –no-register –no-detection –soma-diameter 5 –threshold 3 –ball-xy-size 2 –ball-z-size 7 –ball-overlap-fraction 0.77 –log-sigma-size 0.1 –save-csv –trained-model /trained_network/model.h5

#### Differentiating puncta and diffuse patterns of c-fos staining

To differentiate between punctate and diffuse patterns of *c-fos* staining, we developed a convolutional neural network (CNN) built in Python using the TensorFlow library. The architecture of the CNN is outlined in Extended Data Table 4-1. *c-fos* images from 10 brains were labeled, totaling 2,448 puncta and 1,916 cytoplasmic labels. A training dataset was created by isolating 11 × 11 × 11 pixel cubes centered around each of the labeled cells. The dataset was split 80/20 into a training set and a testing set. The input data was augmented through a series of horizontal and vertical flips, 90° rotations, and 2 pixel horizontal translations to create a total training dataset of 13,706 puncta and 10,724 diffuse labels. No data augmentation was performed on the testing set. The model was trained using an NVIDIA GeForce 3090 GPU for 500 epochs. The batch size was 32, the weight decay rate was 0.0005, and the learning rate was 0.0001. The model achieved an accuracy of 95.3% on the testing set.

#### Brain registration

Image registration was performed using ANTs (Avants et al., 2009). For the nonlinear diffeomorphic step, four parameters were optimized: cross-correlation, gradient step, update field variance in voxel space, and total field variance in voxel space to achieve the best alignment. We chose the parameters based on a combination of qualitative and quantitative measures. Using mutual information (MI; using the measureImageSimilarity function in ANTs with 32 bins) and a binarized segmentation mask, we quantified how well we were able to register the AZBA autofluorescence image to our autofluorescence template (Extended Data Fig. 2-2*A*). We found that most parameters did not make a large difference in MI except total field variance where a value of 0 was superior to 0.5. We got a similar result when we measured the average MI for AZBA registered to individual brains for the 15 min *c-fos* group (Extended Data Fig. 2-2*B*). Thus, we qualitatively determined the best parameters by examining broad alignment of regions like the optic tectum, mamillary bodies, cerebellum, and anterior commissure. These parameters were cross-correlation of 3, gradient step of 0.3, update field variance of 4, and a total field variance of 0. Using these optimized parameters, brain registration was carried out in two steps: first, an average brain template was created, and second, AZBA was registered to this average template.

Before registration, images were downsampled to 4 μm isotropic using brainreg from the BrainGlobe suite of tools (Tyson et al., 2021):

brainreg /path/to/raw/data /path/to/output/directory -v 3.990 0.943 0.943 –orientation sal –atlas azba_zfish_4µm –debug

The average template was generated using 10 autofluorescence images. Initially, nine autofluorescence images were individually brought into the space a single image (template) using the following ANTs command:

antsRegistration –dimensionality 3 –float 1 -o [${AF_sample_1_for_avg_},${ AF_sample_1_for_avg-warped}] –interpolation WelchWindowedSinc -u 0 -r [${AF_template.nii},${AF_sample_1.nii},1] -t Rigid[0.1] -m MI[${AF_template.nii},${AF_sample_1.nii},1,32,Regular,0.25] -c [200 x 200 x 200 x 0,1e-8,10] –shrink-factors 12x8x4x2 –smoothing-sigmas 4x3x2x1vox -t Affine[0.1] -m MI[${AF_template.nii},${AF_sample_1.nii}, 1,32,Regular,0.25] -c [200 x 200 x 200 x 0,1e-8,10] –shrink-factors 12x8x4x2 –smoothing-sigmas 4x3x2x1vox -t SyN[0.3,4,0] -m CC[${AF_template.nii},${AF_sample_1.nii}, 1,3] -c [200 x 200 x 200 x 200, 1e-6,10] –shrink-factors 12x8x4x2 –smoothing-sigmas 4x3x2x1vox –verbose 1

These outputs were then used to create an average image using the “AverageImages” command in ANTs. Next, the autofluorescence image from AZBA was registered to the average template using the following command:

antsRegistration –dimensionality 3 –float 1 -o [${AZBA_to_avg_temp_},${AZBA_to_avg_temp-warped}] –interpolation WelchWindowedSinc -u 0 -r [${avg_template.nii.gz},${AZBA/20180628_AF_average.nii.gz},1] -t Rigid[0.1] -m MI[${avg_template.nii.gz},${AZBA/20180628_AF_average.nii.gz},1,32,Regular,0.25] -c [200 x 200 x 200 x 0,1e-8,10] –shrink-factors 12x8x4x2 –smoothing-sigmas 4x3x2x1vox -t Affine[0.1] -m MI[${avg_template.nii.gz},${AZBA/20180628_AF_average.nii.gz}, 1,32,Regular,0.25] -c [200 x 200 x 200 x 0,1e-8,10] –shrink-factors 12x8x4x2–smoothing-sigmas 4x3x2x1vox -t SyN[0.3,4,0] -m CC[${avg_template.nii.gz},${AZBA/20180628_AF_average.nii.gz}, 1,3] -c [200 x 200 x 200 x 200, 1e-6,10] –shrink-factors 12x8x4x2 –smoothing-sigmas 4x3x2x1vox –verbose 1

To bring the segmentation from AZBA into the space of the template, we used the following command:

antsApplyTransforms -d 3 –float -n NearestNeighbor -i /AZBA/2021-08-22_AZBA_segmentation.nii.gz -r avg_template.nii.gz -o AZBA_to_avg_temp_transformed.nii.gz -t AZBA_to_avg_temp_1Warp.nii.gz -t AZBA_to_avg_temp_0GenericAffine.mat

The newly generated average template image was used as a reference image and was registered onto individual autofluorescence images:

antsRegistration –dimensionality 3 –float 1 -o [${AF_sample_},${AF_sample-warped}] –interpolation WelchWindowedSinc -u 0 -r [${AF_sample.nii},${avg_template.nii.gz },1] -t Rigid[0.1] -m MI[${AF_sample.nii},${ avg_template.nii.gz },1,32,Regular,0.25] -c [200 x 200 x 200 x 0,1e-8,10] –shrink-factors 12x8x4x2 –smoothing-sigmas 4x3x2x1vox -t Affine[0.1] -m MI[${AF_sample.nii},${ avg_template.nii.gz },1,32,Regular,0.25] -c [200 x 200 x 200 x 0,1e-8,10] –shrink-factors 12x8x4x –smoothing-sigmas 4x3x2x1vox -t SyN[0.3,4,0] -m CC[${AF_sample.nii},${ avg_template.nii.gz },1,3] -c [200 x 200 x 200 x 200, 1e-6,10] –shrink-factors 12x8x4x2–smoothing-sigmas 4x3x2x1vox –verbose 1

Finally, segmentation of individual brains was done using the same command as above but applied to the segmentation file as the floating image.

#### C-fos cell counts and network analysis

R (version 4.1.1; R Core Team, 2016) was used for network analysis and to integrate the output from CellFinder with the brain segmentation using the RNifti package (Clayden et al., 2021) to read in the segmentation files. The number *c-fos-*positive cells in each brain were summed excluding white matter and clear labeled regions yielding 143 gray matter regions for analysis. The olfactory bulbs of two brains in the 15 min group did not register properly, so these regions from these two brains were removed from the analysis.

Network analysis was performed using the igraph (version 2.0.2) package (Csardi and Nepusz, 2006). The network was generated by treating the correlation matrix (Fig. 6) as an adjacency matrix. For thresholding, we chose the network density using efficiency cost optimization to maximize the quality function (Fallani et al., 2017):

$$J=\;\frac{E_{g}+E_{l}}{\rho },$$

where $E_{g}$ is the global efficiency, $E_{l}$ is the average of the local efficiency, and ρ is the network density. For the calculations of global and local efficiency, we used a binarized network based on the absolute value of the correlations.

For identifying node roles, we first calculated community structure using the Louvain algorithm (Blondel et al., 2008), and then the within module degree *z*-score:

$$z_{i}=\frac{\kappa_{i}-{\bar{\kappa }}_{s_{i}}}{\sigma_{\kappa_{s_{i}}}},$$

where $\kappa_{i}$ is the number of connections between node i and other nodes in the same community and ${\bar{\kappa }}_{s_{i}}$ is the average of overall nodes in a community; $\sigma_{\kappa_{s_{i}}}$ is the standard deviation of the number of connections in a community. We also calculated the participation coefficient:

$$P_{i}=1-\overset{N_{c}}{\underset{s=1}{\sum }}\;{\left(\frac{K_{\mathrm{is}}}{K_{i}}\right)}^{2},$$

where $N_{c}$ is the number of communities, $K_{\mathrm{is}}$ is the number of connections between node i and all other nodes in community s, and $K_{i}$ is the degree of node i. The definitions of the above equations and the boundaries for the different types of nodes were based on the Guimerà and Amaral (2005).

The small worldness parameter was calculated as described in Humphries and Gurney (2008):

$$\sigma =\frac{\frac{L_{g}}{L_{\mathrm{rand}}}}{\frac{C_{g}}{C_{\mathrm{rand}}}},$$

where $L_{g}$ is the average shortest path length between all nodes of the network, $L_{\mathrm{rand}}$ is the average shortest path length between all nodes in an equivalent random network, $C_{g}$ is the clustering coefficient of the network, and $C_{\mathrm{rand}}$ is the clustering coefficient of an equivalent random network. For random network parameters, we took the average from 1,000 instances of Erdos–Renyi random networks (Erdös and Rényi, 1960) with an equivalent number of nodes and edges as the target network.

#### Statistical analysis

Statistical analysis was done using R. Data were analyzed using 2 × 2 ANOVAs as indicated in the results. For the overall time course *c-fos* data, Dunnett's *t* tests were used to compare all other groups to the home tank control group (time = 0). False discovery rate (FDR; Benjamini and Hochberg, 1995) corrected paired *t* tests at each time point were used for diffuse versus punctate data. For comparing *c-fos* counts across regions between the home tank and 15 min time point, independent samples *t* tests were used with *p* values were corrected using the FDR.

## Results

### Overview of strategy

**Figure 1.**
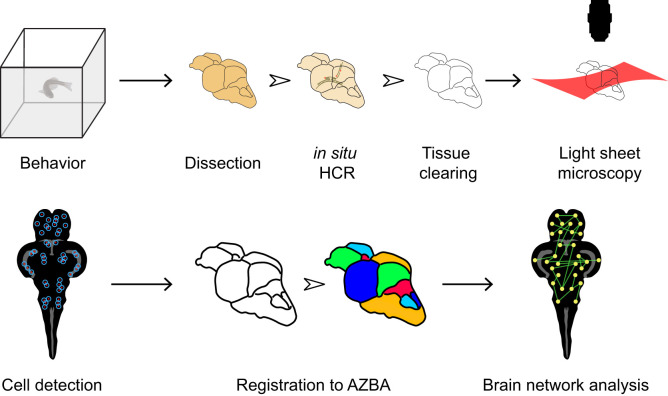
Overview of pipeline for mapping neural activity in adult zebrafish. Following behavior, zebrafish are killed and brains carefully removed. In situ HCR is then used to label *c-fos*. Brains are then cleared using iDISCO and imaged via light-sheet microscopy. Cells are then detected using CellFinder and brains are registered to AZBA. Regional *c-fos* counts are then used to generate brain networks for further analysis. A bench protocol for these methods are available on Dryad; see the code availability statement for a link.

We begin by giving an overview of our strategy for whole-brain activity mapping (Fig. 1) before describing the results of each step in more detail. Following behavior, animals are killed and head fixed in 4% paraformaldehyde overnight. After careful dissection, brains are rendered optically transparent using iDISCO (Renier et al., 2016), which we modified to make compatible with in situ hybridization chain reaction (HCR) for the detection of *c-fos* mRNA (Choi et al., 2018; Kramer et al., 2018; Kumar et al., 2021). Imaging intact cleared brain tissue was done using light-sheet microscopy. To automatically identify *c-fos-*positive cells in the brain, we used the open-source CellFinder package (Tyson et al., 2021) that is part of the BrainGlobe suite of Python-based software tools (Claudi et al., 2020). Finally, to automatically parcellate the brain into individual regions, we used advanced normalization tools (ANTs; Avants et al., 2009) to register autofluorescence images to AZBA (Kenney et al., 2021). The final output of our pipeline is a list of *c-fos*-positive cell counts for each brain region and each animal. This enables the use of a variety of downstream analytic tools; one example we demonstrate here is functional network analysis.

### Automated cell detection

**Figure 2.**
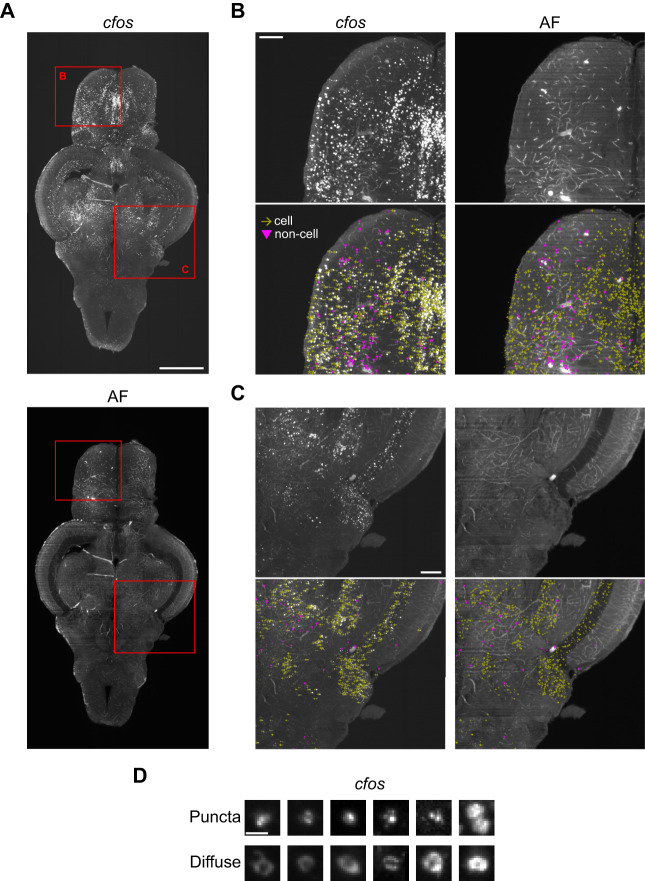
Staining for *c-fos* and identifying *c-fos*-positive cells. ***A***, Adult zebrafish brain stained for *c-fos* (top) and the corresponding autofluorescence image (bottom). Scale bar, 0.5 mm. To see how brains are mounted for imaging, see Extended Data Figure 2-1. ***B***, ***C***, Zoomed in sections of the brain corresponding to red squares in part ***A*** showing *c-fos* staining and autofluorescence with labeling of cells (yellow arrows) and noncells (pink triangles). Scale bars, 0.1 mm. ***D***, Examples of *c-fos* staining characterized as punctate or diffuse. Scale bar, 10 μm.

After in situ HCR, tissue was cleared using iDISCO, which allowed us to use light-sheet microscopy to capture whole-brain images in both the *c-fos* (Fig. 2*A*, top) and autofluorescence channels (Fig. 2*A*, bottom). Detection of *c-fos*-positive cells was done using CellFinder (Tyson et al., 2021), an artificial neural net-based supervised machine learning algorithm. The first step in the cell detection process uses image filtering to detect cell shaped objects in the *c-fos* image. We found parameters that captured *c-fos*-positive cells throughout the entire brain (described in the Materials and Methods section), including areas with cells of different sizes and densities like the telencephalon (Fig. 2*B*) and cerebellum (Fig. 2*C*). Because the cell detection algorithm generated many overlapping cells, we used a custom-written Python script to remove cell candidates that were within 9 μm of one another. We then trained a CellFinder artificial neural network by manually labeling 10,597 cells and 7,303 noncells across five brains. Noncells were unambiguously identified by the presence of a signal in both the *c-fos* and autofluorescence channels, suggesting the presence of background bleeding into the *c-fos* channel. Cells only appeared in the *c-fos* channel. The resulting network achieved over 95% accuracy where the cells and noncells were clearly differentiated across different brain regions (Fig. 2*B*,*C*).

10.1523/ENEURO.0382-24.2025.f2-1 Figure 2-1 Mounting of the zebrafish brain for light sheet imaging. A) Ultraviolet cured resin is first used to form a hemisphere to raise the brain above the platform. B) Zebrafish brain mounted on top of the resin hemisphere. The brain is illuminated with ultraviolet light to make it visible. C) Sample holder where the platform is mounted before being placed in the imaging chamber. D) Sample holder in the imaging chamber of the Ultramicroscope II. Download Figure 2-1, TIF file.

10.1523/ENEURO.0382-24.2025.f2-2 Figure 2-2 Download Figure 2-1, TIF file.

During imaging, we noticed that we had sufficient resolution to differentiate distinct patterns of *c-fos* staining: punctate and diffuse, which likely represent nuclear and cytoplasmic staining, respectively (Fig. 2*D*). This localization of *c-fos* may be an indication of how long ago the cell was active as the mRNA is first transcribed in the nucleus before being shuttled to the cytoplasm for translation. To capture these distinct patterns, we created and trained an artificial neural net on 2,448 examples of punctate and 1,916 examples of diffuse staining. This network achieved >95% accuracy.

### Registration to the adult zebrafish brain atlas

**Figure 3.**
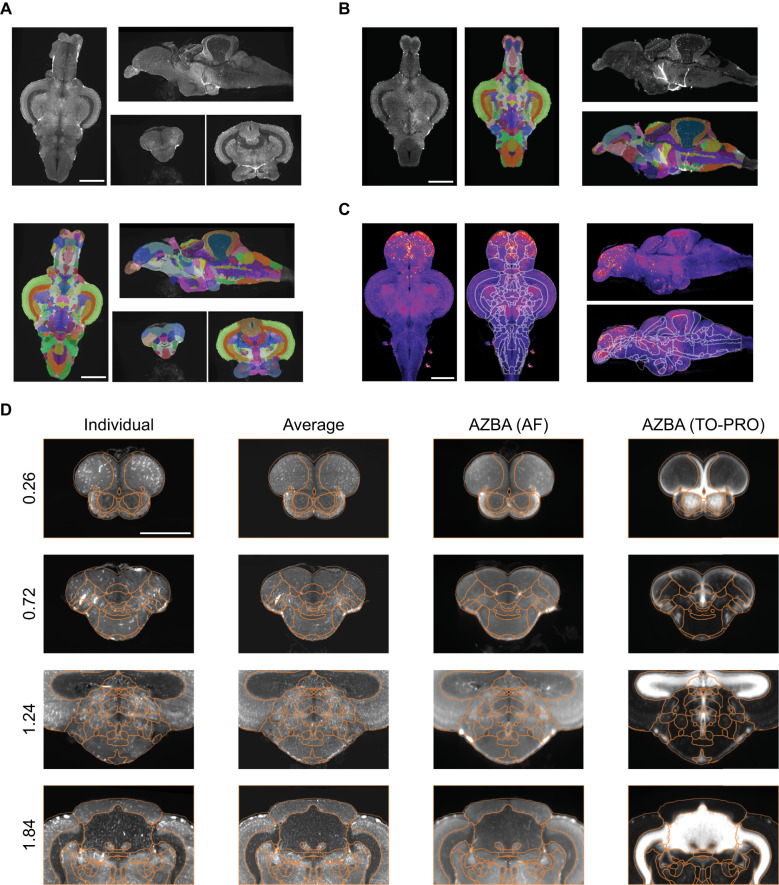
Registration of brain images to AZBA. ***A***, Average image of 10 brains from the present study registered together (top) with segmentation overlay from AZBA (bottom). ***B***, Segmentation from AZBA applied to an individual zebrafish brain. ***C***, An individual *c-fos* brain brought into the space of AZBA with and without segmentation. ***D***, Overlays of segmentation from AZBA in autofluorescence images from an individual brain, the average brain template from the present study, AZBA, and the nuclear TO-PRO stain in AZBA. A quantitative assessment of registration quality can be found in Extended Data Figure 3-1. The numbers on the left indicate distance from anterior most portion of the brain in mm. Scale bars, 0.5 mm.

The adult zebrafish brain contains over 200 regions, making manual segmentation implausible. To automate parcellation of brains into individual regions, we used ANTs (Avants et al., 2009) to register brains to AZBA making use of common autofluorescence images in the atlas and present study. Initially, we attempted to register the autofluorescence image in AZBA directly to individual autofluorescence images, but the results were inconsistent (data not shown). We had more success using an average template that was generated by registering together 10 autofluorescence images from present study (Fig. 3*A*, top). The autofluorescence image from AZBA was then successfully registered to this template brain (Fig. 3*A*, segmentation overlay on bottom). A handful of small anomalies arose from this registration process that we manually fixed using ITK-SNAP (Yushkevich et al., 2019). These arose in parts of the image that tend to be highly variable between individuals, such as where mounting occurs at the ventral hypothalamus and the dorsal sac that extends from the diencephalon. To segment individual brains, we used the transforms from registering the template average autofluorescence brain to individual images (Fig. 3*B*). Using inverse transformations from the registration process, we also brought *c-fos* images into the space of AZBA (Fig. 3*C*). Finally, to demonstrate the quality of our registration, we juxtaposed segmentation from individual and average autofluorescence brains from the present study with the autofluorescence and nuclear stain (TO-PRO) in AZBA (Fig. 3*D*). We included the AZBA nuclear stain because it was the primary image used for segmentation due to its similarity to the cresyl violet stain of the original atlas (Wulliman et al., 1996), and it more clearly delineates regions than the autofluorescence images. Registration was able to successfully segment white matter tracts (e.g., the anterior commissure; Fig. 3*D*, second row); large areas like the cerebellum, optic tectum, and the periventricular gray zone (Fig. 3*D*, bottom row); and numerous telencephalic regions and midbrain nuclei (Fig. 3*D*, first, second, and third rows).

10.1523/ENEURO.0382-24.2025.f3-1 Figure 3-1 Quantitative assessment of image registration. A) Mutual information of the autofluorescence image from AZBA with the average template in the present study. B) Normalized mutual information of the autofluorescence image from AZBA registered to individual brains from the 15-minute *cfos* group. Error bars represent standard error of the mean; n=13. Download Figure 3-1, TIF file.

### Time course for *c-fos* expression

**Figure 4.**
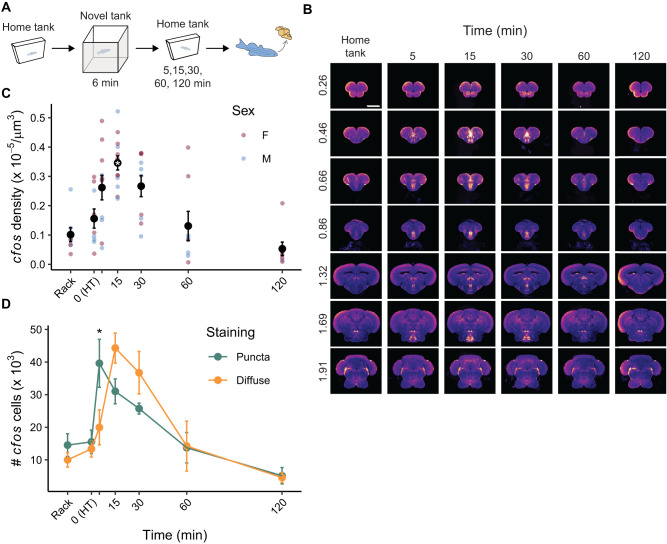
Time course for *c-fos* expression following exploration of a novel tank. ***A***, Experimental design for capturing the time course for *c-fos* expression. ***B***, *c-fos* stained brains from each time point were brought into the space of AZBA and averaged and displayed in the coronal plane. The numbers on the left of image are the distance (in mm) from the anterior most portion of the brain. Scale bar, 0.5 mm. ***C***, *c-fos* cell density from animals taken off the rack, that remained in their home tank (HT), or at different times after exploration of the novel tank (5, 15, 30, 60, or 120 min). *p < 0.05 compared with the HT group using Dunnett's *t* test. Only comparisons with an asterisk were significant. Error bars are standard error of the mean. ***D***, Number of *c-fos* cells classified as punctate or diffuse at each time point. See Extended Data Table 4-1 for the structure of the artificial neural net used for classification. *p < 0.05 difference between the number of puncta versus diffuse stained cells within time point using FDR-corrected *t* tests. Error bars are standard error of the mean. Sample sizes were as follows: rack: female, *n* = 4, male, *n* = 4; HT: female, *n* = 5, male, *n* = 4; 5 min: female, *n* = 6, male, *n* = 5; 15 min: female, *n* = 7, male, *n* = 6; 30 min: female, *n* = 5, male, *n* = 5; 60 min: female, *n* = 4, male, *n* = 4; 120 min: female, *n* = 5, male, *n* = 3.

To effectively map whole-brain activity, we need to know at what point after behavior *c-fos* expression peaks. We exposed fish of both sexes to a commonly used behavioral task, the novel tank test, and killed animals 5, 15, 30, 60, or 120 min following the behavior (Fig. 4*A*). We also had two control groups: (1) fish that were killed immediately after removal from the housing racks and (2) fish that were brought into the behavioral room and killed an hour later, mimicking the habituation to the room we use for fish that were exposed to the novel tank (i.e., time = 0). From the *c-fos* average images at each time point, we observed a large increase in staining between 5 and 30 min (Fig. 4*B*). We also quantified the number of *c-fos*-positive cells using CellFinder and used a 2 × 2 (sex × time) ANOVA for analysis (Fig. 4*C*). We found a large effect of time (*p* < 0.001, *η*^2^ = 0.54), a trend toward a small effect of sex (*p* = 0.07, *η*^2^ = 0.059) and no interaction (*p* = 0.46). The small, but nonsignificant, effect of sex was in the direction of females having slightly higher *c-fos* counts than males. Using a Dunnett's *t* test, we compared all groups with the home tank (HT) control group and found a large increase in *c-fos* cell density at 15 min (*p* = 0.00067, *d* = 2.07). There were no differences at any other time point (*p*'s > 0.05; Fig. 4*C*).

10.1523/ENEURO.0382-24.2025.t4-1 Table 4-1 Architecture of CNN for detecting cytoplasmic and nuclear *cfos* stained cells. Download Table 4-1, DOCX file.

We also examined how the proportion of punctate and diffusely stained cells changed across time (Fig. 4*D*). A cell type × time ANOVA found a large main effect of time (*p* < 0.001, *η*^2^ = 0.40) and no overall effect of cell type (*p* = 0.95). There was also a large interaction between cell type and time (*p* = 0.0082, *η*^2^ = 0.13). FDR-corrected paired *t* tests at each time point found that there were more punctate than diffuse stained cells at 5 min (*p* = 0.048). This trend switched to more diffuse than punctate stained cells at 15 and 30 min, although the differences at these time points were not statistically significant (*p*'s = 0.16 and 0.22, respectively).

### Regions and cell types active during the novel tank test

**Figure 5.**
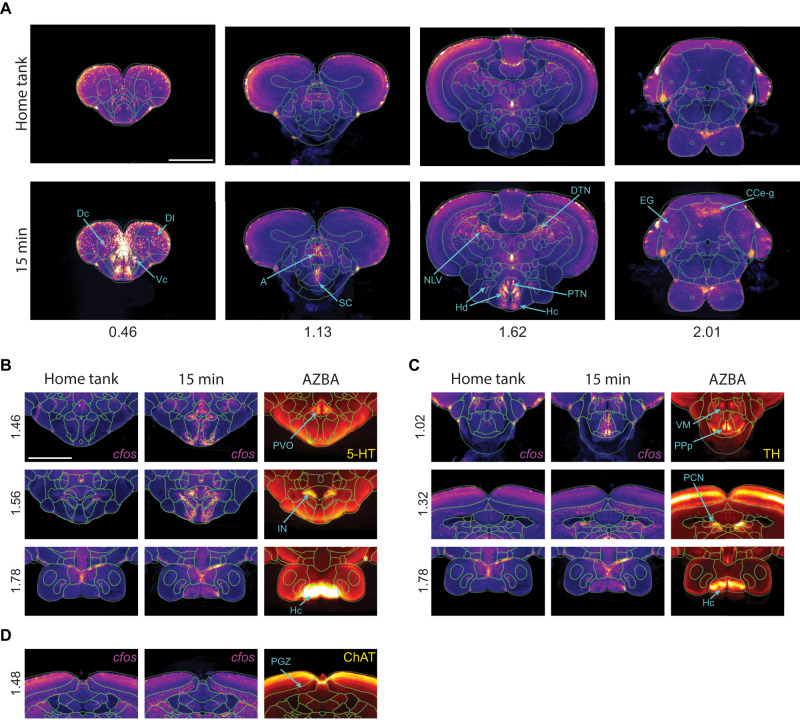
Regions and cell type active during the novel tank test. ***A***, Average c-fos staining from home tank and brains removed 15 min after the novel tank test with a segmentation overlay. Regions indicated are significantly increased compared with the home tank group (FDR corrected *p* < 0.05). Regional abbreviations can be found in Extended Data Table 5-1 and full statistical results in Extended Data Table 5-2. ***B***–***D***, Overlap between *c-fos* expression and neurotransmitter-related stains in AZBA. Regional overlap for (***B***) 5-HT, (***C***) TH, and (***D***) ChAT. Scale bars, 0.5 mm. Numbers on the bottom or left are distance from anterior most portion of the brain in millimeter.

Comparing *c-fos* counts between brains of the home tank and 15 min groups identified 46 regions that had elevated *c-fos* following exposure to the novel tank (Extended Data Table 5-2 with a subset labeled in Fig. 5*A*). In the telencephalon, regions with elevated activity included parts of both the pallium [lateral (Dl) and central zones (Dc), the anterior part of the nucleus of the olfactory tract (nLOT-a), and the medial division of the bed nucleus of the stria terminalis (BSTm)] and subpallium [lateral (Vl) and central nuclei (Vc)]. Several thalamic nuclei were also upregulated, such as the anterior (A) and intermediate (I) thalamic nuclei suggesting strong engagement of sensory systems during exploration. The hypothalamus, which is central to endocrine regulation, had increased *c-fos* in the caudal (Hc) and dorsal (Hd) zones as well as the lateral hypothalamus. Finally, several regions of the cerebellum also had high levels of *c-fos* during exploration of the novel tank, such as the granular and molecular layers of the cerebellar corpus (CCe-g/m) and the granular eminence (EG).

10.1523/ENEURO.0382-24.2025.t5-1 Table 5-1 List of abbreviations for grey matter regions. Download Table 5-1, DOCX file.

10.1523/ENEURO.0382-24.2025.f5-2 Table 5-2 Comparison of *cfos* counts in hometank control and animals euthanized 15 minutes after the novel tank test. Download Table 5-2, CSV file.

AZBA contains several stains that can be used to identify different cell types across brain regions such as 5-hydroxytryptamine (5-HT), tyrosine hydroxylase (TH), and choline acetyltransferase (ChAT; Kenney et al., 2021). To determine if exposure to a novel tank results in the activation of regions containing these neuronal cell types, we looked for overlap between the stains in AZBA and elevated *c-fos* (Fig. 5*B*–*D*). For regions expressing 5-HT (Fig. 5*B*), we saw an increase in *c-fos* in the paraventricular organ (PVO), intermediate nucleus (IN), and caudal zone of the periventricular hypothalamus (Hc). For TH, we saw overlap in the ventromedial thalamic nucleus (VM), the posterior part of the parvocellular preoptic nucleus (PPp), paracommissural nucleus (PCN), and Hc (Fig. 5*C*), although only the elevation of *c-fos* in PCN and Hc was statistically significant (Extended Data Table 5-2). Finally, for ChAT, we saw overlap in the paraventricular gray zone of the optic tectum (PGZ; Fig. 5*D*), however, this elevation did not reach significance. One reason we may see differences in *c-fos* average images that did not reach statistical significance is the lack of power; with our current number of observations (*n*'s = 9–13), we are only able to detect very large effect sizes (cohen's *D* > ∼1). Although we can see an overlap at the regional level, our findings are only tentative because the *c-fos* and antibody-stained images come from separate brains, so we cannot make claims at the cellular level. Nonetheless, this demonstrates how our approach can be used to generate hypotheses about roles different neurotransmitters may play in an underlying a behavior.

### Brain network analysis

**Figure 6.**
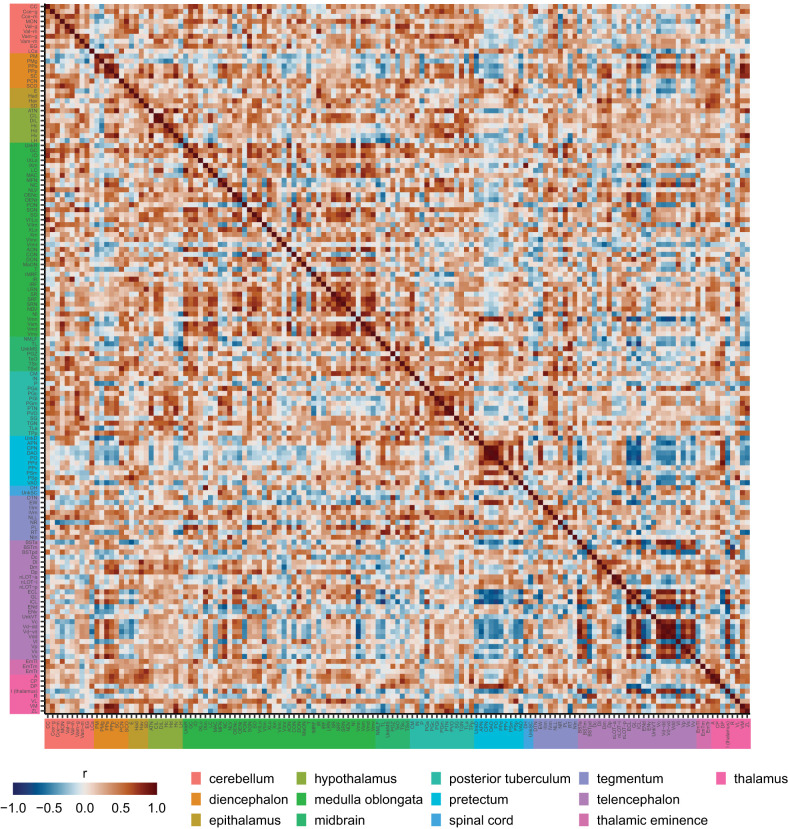
Correlation matrix of *c-fos* activity across the zebrafish brain. Entries in the matrix are Pearson’s correlations between brain regions across animals killed 15 min after the novel tank test. Regions are organized based on common ontological levels. Regional abbreviations and ontological levels can be found in Extended Data Table 5-1.

**Figure 7.**
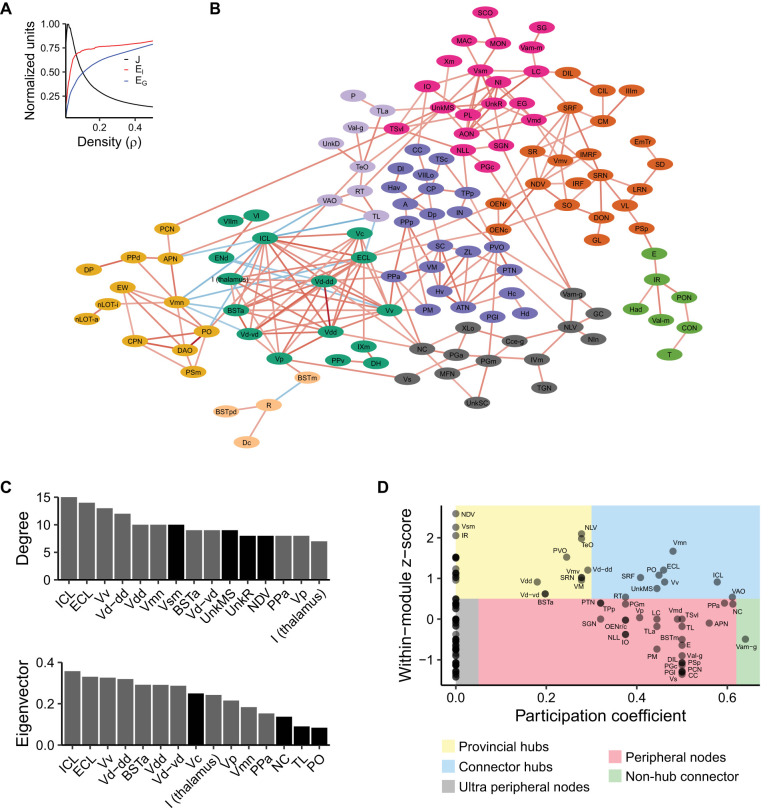
Analysis of the functional brain network active during the novel tank test. ***A***, Efficiency–cost optimization for different network densities. *J*, quality function (see Materials and Methods); *E_l_*, local efficiency; *E_G_*, global efficiency. ***B***, Network filtered at a density of 2.5%. Connections between nodes represent suprathreshold correlations from Figure 6. Color of connections represents the strength (darker means higher absolute value) and direction (red, positive; blue, negative) of the correlation. Node colors correspond to communities. Regions not in the giant component are not shown. ***C***, Degree and eigenvector centrality for the top 15 brain regions. Gray bars are those regions that are in the top 15 for both degree and eigenvector centrality. ***D***, Identification of the role that each node plays in the network based on within module degree *z*-score and participation coefficient.

We used functional network analysis to gain insight into the organization of brain activity that underlies exploration of a novel tank (Wheeler et al., 2013; Vetere et al., 2017; Pinho et al., 2023). Using *c-fos* counts from the 15 min time point, we computed the correlated activity between all 143 gray matter regions across animals (Fig. 6). To filter the correlation matrix to generate a network, we used efficiency cost optimization where the network density is chosen such that it balances the inclusion of edges to increase global and local efficiency against the putative cost of including additional connections (Fallani et al., 2017). We found a density of 2.5% maximized the efficiency cost optimization quality function (Fig. 7*A*). This resulted in a network with 256 edges and an average degree of 3.6, which is consistent with functional brain networks from other species using different imaging modalities (Fallani et al., 2017). This network also exhibited small world properties: its average shortest path length between nodes was 5.3, similar to the average path length from equivalently dense random networks (3.9). Our network also had much higher clustering than random networks (0.42 vs 0.025). This yielded a small world coefficient >1 (12.6) indicating the expected small world property (Humphries and Gurney, 2008). We also computed degree and eigenvector centrality for each node to uncover brain regions that may play outsized roles in the network, uncovering several regions that were in the top 15 for both measures (Fig. 7*C*). These included several telencephalic regions, particularly from the subpallium, such as the ventral nucleus of the ventral telencephalon (Vv), the dorsal and ventral zones of the ventral telencephalon (Vd-dd and Vd-vd), and the dorsal most zone of the ventral telencephalon (Vdd). A handful of regions outside the telencephalon were also high in centrality, such as the intermediate thalamic nucleus (I), the anterior part of the parvocellular preoptic nucleus (PPa), the mesencephalic trigeminal nucleus (Vmn), and two regions in the olfactory bulb [external (ECL) and internal (ICL) cellular layers].

To identify the community structure of the network, we used the Louvain algorithm (Blondel et al., 2008), which identified nine clusters (Fig. 7*B*). Using this community structure, we categorized the roles that different nodes play in interconnecting different parts of the network (Guimerà and Amaral, 2005): provincial hubs (highly connected within its community, but not between communities), connector hubs (highly connected both within and between communities), peripheral nodes (low connectivity within and between communities), and nonhub connectors (low connectivity within a community, but high between communities). The Vv, Vmn, and PPa, which were identified as important based on centrality measures, are connector hubs (or nearly so for the PPa). The PPa and Vv interconnect the module dominated by regions of the ventral telencephalon with other parts of the preoptic area (SC and PPp), thalamus (VM, CP, and ZL), and hypothalamus [ATN (anterior tuberal nucleus), Hv (ventral zone of the periventricular hypothalamus), Hc, and Hd]. Interestingly, Vmn has a lot of inverse connections with parts of the telencephalon and provides a link to a cluster dominated by pretectal regions like the DAO (dorsal accessory optic nucleus), PSm (magnocellular superficial pretectal nucleus), CPN (central pretectal nucleus), PO (posterior pretectal nucleus), and PPd (dorsal part of the periventricular pretectal nucleus). Finally, two olfactory bulb regions [the internal (ICL) and external (ECL) layers] were high in centrality and identified as hubs. Both the ICL and ECL had most of their connections with the subpallium. The ICL also had several negative connections with the pretectal cluster described above, and the ECL connected to the octavolateralis neurons. Thus, our network analysis points to the PPa, ventral telencephalon, olfactory bulbs, and Vmn as likely playing an important role in regulating behavior during exploration of a novel tank.

## Discussion

In the present study, we introduce a pipeline for performing whole-brain activity mapping in adult zebrafish. Our pipeline combines several recently developed tools: a digital brain atlas for adult zebrafish (Kenney et al., 2021), registration using ANTs (Avants et al., 2011), machine learning for automated cell detection (Tyson et al., 2021), tissue clearing (Renier et al., 2014), light-sheet microscopy (Reynaud et al., 2015), and in situ HCR (Choi et al., 2018) for detecting *c-fos*. All computational tools are open source and free to use. Furthermore, to aid in the implementation of this pipeline, we have included a bench protocol. The primary stumbling blocks for implementing this pipeline are likely to be access to a light-sheet microscope for whole-brain imaging, and sufficient computational power for training and applying the registration and CellFinder machine learning algorithms. The former issue is partly mitigated by the increased availability of light-sheet microscopes, particularly in core facilities. Access to computational resources can be addressed by using tools like Google Colaboratory (Bisong, 2019) or high performance computing facilities available at many institutions.

### *C-fos* to capture whole-brain activity

We captured neural activity using in situ HCR to detect *c-fos* mRNA. We chose this approach for several reasons: (1) there are a paucity of antibodies for detecting c-fos protein in zebrafish, none of which are known to work in whole-mount tissue-cleared samples; (2) in situ HCR probes are small (∼150 bp), which easily penetrates chunks of intact tissue like the adult zebrafish brain; and (3) *c-fos* is one of the most widely used markers of neural activity due to low background, high signal-to-noise, and good temporal resolution that arises from its autoinhibition of transcription (Lucibello et al., 1989; Chung, 2015). Furthermore, *c-fos* has been successfully used to capture neural activity in both adult and larval zebrafish (Baraban et al., 2005; Lau et al., 2011; Ruhl et al., 2017; Shainer et al., 2023). The findings in the present study further support our rationale: we saw even penetration of *c-fos* staining throughout the brain (Figs. 2*C*, 4*B*) and the levels of background *c-fos* staining were low, with an ∼3.5-fold increase in *c-fos* density 15 min following behavior compared with animals removed directly from their housing racks (Fig. 4*C*). The increase in *c-fos* was also tightly coupled to the behavior, peaking 15 min after exposure to the novel tank before decreasing to baseline by 60 min. Interestingly, if we look at only cells that have more puncta like staining, we see the increase begins as soon as 5 min after the behavior (Fig. 4*D*). We believe that these puncta represent the initial burst of transcription in the nucleus. The shift from more puncta-like to more diffuse-like staining over 10–15 min is consistent with the time it would take for mRNA to be transcribed and shuttled from the nucleus to the cytoplasm (Oeffinger and Zenklusen, 2012). However, additional experiments are necessary to more definitively draw this conclusion. The time to maximal *c-fos* we observed is faster than is seen in rodents, where it is often found to peak at 30 min poststimulation (Ding et al., 1994; Kovács, 1998; Guzowski et al., 2001; Zangenehpour and Chaudhuri, 2002). The reason for this time difference between zebrafish and rodents is unclear. Nonetheless, it emphasizes the importance of performing time course analysis when establishing new methods for brain mapping in different species.

Other markers of neural activity have gained traction in recent years in zebrafish, such as the phosphorylated forms of ribosomal protein S6 (pS6) and extracellular regulated kinase 1/2 (pERK1/2). Our data suggests that *c-fos* as an activity marker compares favorably with these options. For pS6, the signal-to-noise ratio is comparable with what we see for *c-fos*, with an ∼2–4-fold increase over baseline both in vivo in zebrafish (Butler et al., 2018; Scaia et al., 2022; Parada et al., 2024) and in vitro neuronal cell culture (Kenney et al., 2015). However, the time course of elevated pS6 is notably slower, taking an hour or more to peak (Kenney et al., 2015; Parada et al., 2024) compared with 15 min for *c-fos* (Fig. 4*C*). In contrast, pERK1/2 activity peaks quickly, within 2–5 min, but the signal-to-noise ratio is ∼0.5–1, lower than *c-fos* (Randlett et al., 2015; Venincasa et al., 2021). This low signal-to-noise ratio likely arises from higher background levels of pERK due to the wide variety of cellular processes that it regulates (Cargnello and Roux, 2011). Thus, the best choice of stain depends on the behavioral paradigm. Large, rapid responses to brief behavioral stimuli are best captured by pERK. However, more subtle responses may be missed due to the low signal-to-noise ratio. S6 phosphorylation excels at capturing long-lasting steady-state neural activity, as suggested by Maruska et al. (2020), and would be ideal for behaviors lasting 30 min or more. c-fos represents a middle ground that is ideal for capturing neural activity from behaviors lasting on the order of 5–10 min, like the novel tank test used in the present study.

### Registration to AZBA to identify cell types

We were able to successfully register our brains to AZBA using ANTs (Avants et al., 2009). To do so, we first used ANTs to make an average template from our images by registering nine brains to a single brain before averaging them together. The autofluorescence image in ABZA was then registered to this average template, yielding excellent results (Fig. 3). We chose this method because we found that registering the autofluorescence image from AZBA to individual brains gave inconsistent results. This is likely because the autofluorescence image in AZBA is also an average of many brains (Kenney et al., 2021). We chose ANTs because the nonlinear symmetric diffeomorphic image registration it employs has been found to be one of the best algorithms for 3D image registration (Klein et al., 2009; Murphy et al., 2011). The tool is also well documented and straightforward to use. Finally, ANTs have recently grown in popularity for image registration in larval zebrafish (Marquart et al., 2017; Shainer et al., 2023), which provided a starting point for identifying the best parameters for registration in our samples.

Following registration to AZBA, we were able to identify regions, and potential neuronal cell types, whose activity were increased following exposure to a novel tank (Fig. 5). We found that several regions containing high levels of 5-HT were active during behavior, such as the PVO, IN, and Hc. Consistent with this, several papers have implicated 5-HT as contributing to exploration of a novel tank using pharmacological approaches (Wong et al., 2010; Maximino et al., 2013; Nowicki et al., 2014; Beigloo et al., 2024). Similarly, there was overlap in *c-fos* activity in several regions that express tyrosine hydroxylase (VM, PPp, PCN, and Hc), implicating these populations of dopaminergic neurons in novel tank behavior (Kacprzak et al., 2017; Nabinger et al., 2023). Of the *c-fos*-positive cells that overlap with TH, our network analysis suggests that the PPp may be of particular importance in regulating exploratory behavior because it has a direct connection to the PPa region, which ranks high in both eigenvector and degree centrality (Fig. 7*C*), and connects to the thalamic VM region, another area high in TH expression. This suggests that the PPp and VM may act in concert to mediate the effects of the dopaminergic system on exploration. However, one important caveat to these interpretations is that we are comparing averaged *c-fos* images to averaged neurotransmitter-related stains in AZBA, and thus we cannot definitively identify the specific cell types that are active. This would require costaining of brains with both *c-fos* and cell-type markers.

### Novel tank functional brain network

Using our whole-brain mapping data, we generated an adult zebrafish novel tank test functional brain network. The novel tank test is one of the most widely used behavioral tests in adult zebrafish, commonly used to study exploratory and anxiety-related behaviors (Spence et al., 2006; Blaser et al., 2010; Luca and Gerlai, 2012; Kalueff et al., 2013; Rajput et al., 2022). Our functional network analysis identifies several key regions that are engaged during exploration of a novel tank (Figs. 6, 7). In particular, the medial portion of the ventral telencephalon stands out, where several subregions (the Vv, Vd-dd, Vdd, Vc, Vd-vd, and Vp) rank highly on at least one measure of centrality (Fig. 7*C*). These regions are also highly interconnected, a fact that is clear from both the correlation matrix (Fig. 6) and the community they form in the network (Fig. 7*B*, dark green). Based on molecular markers, these regions of the subpallium are thought to correspond to the mammalian subpallial amygdala (i.e., the central and medial amygdala) and basal ganglia (Porter and Mueller, 2020; Mueller, 2022). In mammals, these brain regions have been found to be important for a wide range of behaviors, from defensive, anxiety-related, and social behaviors to motor control (Grillner and Robertson, 2016; Fadok et al., 2018; Raam and Hong, 2021). Our findings that the ventral telencephalon appears to be strongly engaged during the novel tank test makes sense given that novelty and exploration would be expected to engage circuits involved in decision-making, emotional regulation, and muscle coordination.

In examining how the regions of the ventral telencephalon interact with the rest of the brain, a few interesting trends emerge. Notably, the interaction of ventral telencephalic regions with many other communities is anticorrelated (Fig. 7*B*, gold and light purple). This suggests the presence of strong inhibitory connections between the medial ventral telencephalon and other parts of the brain. Consistent with this interpretation, the ventral telencephalon has been found to contain a substantial number of inhibitory GABAergic neurons (Porter and Mueller, 2020). Our network analysis suggests that these inhibitory connections are most likely present between the ventral telencephalon and the Vmn (mesencephalic nucleus of the trigeminal nerve), ENd (entopeduncular nucleus in the lateral portion of the ventral telencephalon), and from the BSTm (bed nucleus of the stria terminalis, medial portion in the dorsal telencephalon) to R (rostrolateral nucleus in the thalamus). However, given that our findings are correlational in nature, techniques like tract tracing and direct manipulation would be needed to determine if these interactions are due to direct connections or are mediated by intermediate regions.

Our network analysis also identified the PPa as a region of high importance. The PPa was high in both eigenvector and degree centrality (Fig. 7*C*) and was ranked highly for participation coefficient and above average for within module *z*-score (Fig. 7*D*). In examining its place in the network (Fig. 7*B*), the PPa interconnects with several regions of the ventral telencephalon and, working in concert with the PPp, mediates their interactions with parts of the network that contain thalamic and hypothalamic regions (Fig. 7*B*, dark purple cluster). To our knowledge, the correspondence between the PPa and PPp in teleosts and tetrapods has not been determined. Based on the expression of neuropeptides, like oxytocin and arginine vasopressin, parts of the PPp are thought to be homologous to the supraoptic nucleus in mammals (Herget et al., 2014). In larval zebrafish, the preoptic area has been implicated in behaviors such as navigation, thermoregulation, and stress reactivity (Corradi et al., 2022; Palieri et al., 2024). However, the preoptic area in larval zebrafish cannot be differentiated into subregions like the PPa and PPp due to a lack of cytoarchitectural boundaries (Herget et al., 2014). This makes it unclear as to what specific regions in the adult would subsume the functions identified in larval animals. Future work should determine the role that these different subregions might play in different aspects of exploration and anxiety-like behavior in adult zebrafish.

Unexpectedly, two olfactory bulb regions (ECL and ICL) were high in centrality (Fig. 7*C*) and identified as connector hubs (Fig. 7*D*). Exposure to novelty elicits a combination of exploratory and anxiety-like behaviors and fish may be engaging their olfactory system as they assess the new environment. The correlated activity between the bulbs and many other brain regions likely arises through their extensive projections to the dorsal and ventral telencephalon, habenula, and posterior tuberculum (Miyasaka et al., 2014, 2009). Neural activity in the olfactory bulbs is complex. Activity is not only elicited by incoming sensory information but sculpted by centrifugal projections from other brain regions, like the posterior zone of the dorsal telencephalon (Kermen et al., 2020), and neuromodulators like dopamine, serotonin, acetylcholine, and neuropeptide Y (Byrd and Brunjes, 1995; Clemente et al., 2004; Edwards et al., 2007; Schweitzer and Driever, 2009; Bundschuh et al., 2012; Kawai et al., 2012; Kenney et al., 2021). The importance of the olfactory bulbs in regulating activity across the brain makes sense given that reduction in their function has been associated with depression in humans (Croy and Hummel, 2016) and olfactory bulbectomy in rodents has been used to model mood disorders for over 40 years (Leonard and Tuite, 1981). Indeed, removal of the olfactory bulbs causes many structural changes throughout the brain (Song and Leonard, 2005). Taken together with the present data, it may be that the olfactory bulbs in zebrafish are also instrumental to the functioning of the brain during the exploration of a novel environment.

The only prior work examining the neural basis of exploratory behavior in fish is in larval stage animals where the regions underlying spontaneous locomotion were identified (Dunn et al., 2016). The most prominent region associated with spontaneous turn patterning in larval fish was an anterior portion of the hindbrain. Interestingly, in our novel tank functional brain network, we found several regions in a similar part of the brain with high centrality, the trigeminal nuclei (Vsm and Vmn); however, the exact relationship between these areas in the adult and larval brain is unclear. In larval fish, the preoptic area was also found to be weakly correlated with turning (Dunn et al., 2016), echoing our findings that the PPa appears to be a key region regulating exploratory behaviors. This overlap in the present work in adults and prior work in larval fish suggests that similar neural mechanisms may be at play at these two distinct life stages.

### Caveats and limitations

An important caveat of the present work is that the functional brain network (commonly referred to as a “functional connectome” in human studies) reflects statistical, not physical, relationships between brain areas. Regional covariation may be due to a physical connection or mediation by one or more intermediate regions. This approach to understanding patterns of brain activity is a mainstay in human functional neuroimaging (Bullmore and Bassett, 2011) and is increasingly used in rodents (Wheeler et al., 2013; Ben-Ami Bartal et al., 2021; Verpeut et al., 2023) where it is able to predict regional involvement in behavior (Vetere et al., 2017). In zebrafish, functional connectivity approaches have been used to measure activity in a predefined set of regions in adults, providing insight into the patterns of brain activity important for social behaviors (Akinrinade et al., 2023; Pinho et al., 2023). Our approach builds on this prior work by enabling the capture of neural activity across the entirety of the brain.

One limitation of the present work is our use of iDISCO for tissue clearing. We chose iDISCO because it results in rapid and robust clearing that has been found to be compatible with multiple staining methods, like in situ HCR (Kramer et al., 2018; Kumar et al., 2021). Furthermore, AZBA was created using iDISCO (Kenney et al., 2021), and we wanted to ensure our autofluorescence images used for registration matched as closely as possible to those in AZBA. However, iDISCO requires imaging in an organic solvent, dibenzyl ether, and thus is not compatible with the objectives of many microscopes. Here, we used the UltraMicroscope II, which was specially designed for use with such solvents (Dodt et al., 2015). The use of other light-sheet microscopes may require the building of chambers to prevent the objectives from coming into contact with the solvents, as proposed when iDISCO was first described (Renier et al., 2014). Nonetheless, with the decreasing cost and wider availability of light-sheet microscopes in core facilities, our approach is likely within reach of many scientists.

### Summary

The present study provides an open-source framework for performing whole-brain mapping in adult zebrafish. This work also yielded the first description of brain activity that underlies the novel tank test, suggesting the ventral telencephalon may play an important role in one of the most widely used behavioral tasks in adult zebrafish. Taken together, we anticipate that our pipeline will help generate insights into the principles of brain function by enhancing the utility of adult zebrafish as a model organism.

## Data Availability

Data, code, and a bench protocol are available at Dryad: https://doi.org/10.5061/dryad.k3j9kd5js

## Synthesis

Reviewing Editor: David Schoppik, New York University Grossman School of Medicine Decisions are customarily a result of the Reviewing Editor and the peer reviewers coming together and discussing their recommendations until a consensus is reached. When revisions are invited, a fact-based synthesis statement explaining their decision and outlining what is needed to prepare a revision will be listed below. The following reviewer(s) agreed to reveal their identity: Armin Bahl.

Major:

1. We feel that the technical novelty of the current paper needs to be put into clearer context and aligned with the other studies showing adult brain manipulation. The same for biological novelty. For example, at lines 335-336 the authors state they created the 'first novel tank functional connectome'. A significant number of studies have investigated novel environments, although admittedly less in adults. However, we felt that with only cfos imaging and no circuit tracing, functional imaging, etc. claiming insights into a connectome is an overstatement.

2. Line 60. The authors state 'over the past two decades, zebrafish have started to contributing to our understanding of the brain'. The use of zebrafish in neuroscience predates the year 2000 and

here the authors only cite references from 2020. A bit more careful consideration of the model and prior work would help to make a more impactful argument for the current study.

re: cfos

1. Other groups have used cfos HCR and would be worthwhile studies to mention and cite.

2. In multiple figures the authors claim cfos labeling overlaps with brain regions and markers yet do not show the overlap from registered images. This should be included.

3. The images shown for nuclear vs cytoplasmic cfos are not convincing and some labeling validation for these claims are necessary.

4. The authors describe the euthanasia protocol by placing animals for 5 min in ice-cold water. Could this treatment not lead to a decrease in c-fos expression levels? A comment about this potential problem, or why the authors may not consider this to be problematic, should be added to the methods part.

5. The authors should discuss the time course experiments of c-fos expression more rigorously. It is already quite detailed and good, but the fact that c-fos expression decays again after 15 minutes does not have to imply that c-fos can not accumulate even more - it may also simply mean that fish get habituated to the novel tank, and neuronal activity in the areas starts to decay again.

re: Methods

1. The authors say that they modified the iDisco protocol from the original version to be compatible with HCR FISH. Please explain briefly what those modifications are and why they think it mattered. In a similar vein, the authors may also discuss why they chose iDisco clearing and not one of the other existing methods.

2. Due to its toxicity, iDisco requires the usage of specialized enclosed chambers and optics, making this clearing method quite complex for many labs. The authors only briefly say: "Samples were mounted on the sample holder using an ultraviolet cured resin (NOA 61, Norland Products, Jamesburg, NJ) with a refractive index (1.56) that matched DBE." It would be nice to expand on this, helping readers to decide whether iDisco clearing and HCR FISH would indeed be a useful approach in their labs. Can the authors add an image of the sample holder inside the light sheet microscope to the supplement?

3. How do the authors exclude that the clearing treatment itself induces the diversity of mRNA distributions (nuclear vs. cytosolic)? Can the authors provide a dataset of a non-cleared fish from their light-sheet system or from the published literature that shows nuclear-localized mRNA as well? In Figure 2d, how do they know that the c-fos is clustered inside the nucleus, and not just somewhere at a specific location in the cytosol? Do they have an indication of the location of the cell body from the auto-fluorescent images or a DAPI staining?

4. The use of autofluorescence as a means for registration is potentially interesting yet needs some more evidence to be convincing. The authors even mention challenges with this approach. Showing examples of alignment of the autofluorescence channel, reliable alignment of some neuroanatomical region, and providing some quantification for efficacy would be a significant improvement, especially as this is one of the novel elements of the manuscript.

Minor issues:

1. Line 117. What is the rationale for removing cell candidates within 9um of each other and what impact could this have on analysis/interpretation.

2. Figure 4. The use of an ANOVA for euthanasia time &sex needs further explanation. Were sex differences considered and/or relevant? Was the sex proportions kept consistent across groups?

3. In various parts of the manuscript the use or testing of various species is mentioned yet may be overstated without additional testing as overall brain size, lipid composition, and numerous other factors may not allow the zebrafish techniques to be transferred. This may be worth limiting to a discussion point.

4. Specifically mentioning the amgydala in the abstract is somewhat distracting as it never becomes a significant point again.

5. Line 69. Does 'this behavioral repertoire' refer to adult behavior?

6. In the methods it may be worth combining HCR labeling and clearing. As is the timing and combination of these two techniques is unclear and would seem to be important for another group to perform these techniques.

7. Line 488 sheet numerical aperture of XX?

8. It would be nice to add information about which statistical test was used in Figure 4 (as described in the methods, this should be a Dunnet's t-test). What do the confidence intervals indicate in Figure 4? (std?). Please add this information to the legend. There is only one star (0 vs. 15 min). Does this mean all other tests were not significant? Please add a note in the legend about this point.

9. Probably it's the review version of the document, but in the final paper, a higher resolution version of Figure 2 would be good.

10. The usage of two color maps in Figure 6 is confusing. Could the authors label the brain region name initials in a color based on the super-region, on both the x and y axis? Or use thin colored lines on both axes? This may be a better way to allow one to identify which brain region is (anti)correlated with which brain region.

## Author Response

December 24, 2024 Dear Dr. Schoppick, Thank you for taking the time to consider our manuscript entitled "Whole-brain mapping in adult zebrafish and identification of a novel tank test functional connectome" for publication in eNeuro. We thank the reviewers for their thorough review of our paper and making several useful suggestions that have significantly strengthened the manuscript. We would also like to note that we have uploaded our supplemental data to Dryad. It can be accessed at the following url: http://datadryad.org/stash/share/c_CkemOLrkiYucQsdPhSWxNVREB0H6MNqerFlaD3g3E Below, we have outlined our response to reviewer comments (in italics). Alternations to the manuscript in response to reviewer comments are highlighted.

Major comments "1. We feel that the technical novelty of the current paper needs to be put into clearer context and aligned with the other studies showing adult brain manipulation. The same for biological novelty. For example, at lines 335-336 the authors state they created the 'first novel tank functional connectome'. A significant number of studies have investigated novel environments, although admittedly less in adults. However, we felt that with only cfos imaging and no circuit tracing, functional imaging, etc. claiming insights into a connectome is an overstatement." We have now added several elements to put the current paper in a broader context. For example, in the discussion section. We mention how other groups have successfully used cfos in both adult and larval brains (lines 620-622). We also mention that others have generated functional networks in adult zebrafish (lines 778-782). However, we could not find another study examining the patterns of brain activity during exploration of a novel environment. The closest we found was a study in larval fish by Dunn and colleagues (2016) where they examined spontaneous locomotor activity; we have added a discussion of this paper and how it compares to our results (lines 759-769). Nonetheless, we removed any references to our paper being the 'first novel tank functional connectome'.

With respect to the last point that we are not doing functional imaging and thus cannot claim that we are identifying a connectome, we respectfully disagree with the reviewers. Firstly, given that cfos is a measure of neuronal activity, imaging cfos is a 'functional imaging' approach. Indeed, it is categorically identical to the widely used pERK1/2 that is common in larval zebrafish (e.g., Randlett et al, 2015; Venincasa et al 2021), discussed in lines 640-657. These methods are accepted ways to infer functional relationships between brain activity and behavior. Secondly, we used the term 'functional connectome' to refer to the network we've identified, which indicates that we're capturing statistical relationships between brain regions as opposed to an 'anatomical connectome' that captures physical relationships. Our method of generating a functional connectome using activity markers like cfos has been successfully used in both rodents and zebrafish (Ben-Ami Bartal et al, 2021; Verpuet et al, 2023; Wheeler et al, 2013; Akinrinade et al, 2023; Pinho et al, 2023). Indeed, work in mice has found that this approach is able to more accurately predict brain-behavior relationships than looking at changes in cfos levels (Vetere et al, 2017). To make this point clearer, we have added a paragraph to the discussion discussing how functional connectomes capture statistical, not physical, relationships between regions and mentioning the other work in rodents and fish that have used this approach (lines 772-782). "2. Line 60. The authors state 'over the past two decades, zebrafish have started to contributing to our understanding of the brain'. The use of zebrafish in neuroscience predates the year 2000 and here the authors only cite references from 2020. A bit more careful consideration of the model and prior work would help to make a more impactful argument for the current study." We appreciate the reviewers pointing this out. Admittedly, we were a bit myopic in our citations, focusing on reviews of adult zebrafish behavior. We have now expanded the citations to include older citations in neurodevelopment (Lines 60-63).

Re:cfos "1. Other groups have used cfos HCR and would be worthwhile studies to mention and cite." We thank the reviewers for pointing out this oversight. We have now included several of these references (lines 620-622). "2. In multiple figures the authors claim cfos labeling overlaps with brain regions and markers yet do not show the overlap from registered images. This should be included." We thank the reviewers for this comment as it pushed us to come up with a better way to visually demonstrate the accuracy of our registration by using segmentation outlines in addition to the overlays. More specifically, we have added outlines of the registered segmentation in Figure 3C and added additional images juxtaposing images from the present study with AZBA in Figure 3D. We also added outlined cfos images to figure 5A. This also prompted us to add additional analysis looking at regional differences in cfos levels (lines 505-516; extended file 5-1) to accompany the figure. We believe these images more clearly demonstrate the high quality of the registration we achieved and is a significant improvement to the manuscript.

For the overlap between cfos staining and markers (Figure 5B-D) we also included outlines. We did not generate overlays between the markers and cfos images because they are averages from separate brains as opposed to images of different channels from the same brain. We felt that an overlay may incorrectly lead the reader to believe the images were from the same brains. We feel that the outlines clearly convey that there is overlap in cfos and markers in these regions. However, we are clear in interpreting this as suggestive, and that a more definitive determination of overlap would require co-staining in the same brains (lines 530-534 and lines 686-689). "3. The images shown for nuclear vs cytoplasmic cfos are not convincing and some labeling validation for these claims are necessary." We do not have any way to independently validate the claim that these are nuclear. Early on in this project we attempted to image a nuclear stain (e.g., DAPI and Hoechst). However, because these are stimulated by UV light, which has a short wavelength that absorbs more rapidly, it does not evenly penetrate the tissue despite the clearing. They also tend to bleed into the 488 channel, which would interfere with image registration.

We have now changed the language to refer to 'puncta' and 'diffuse' staining throughout the manuscript (e.g., Figure 4D) for the cfos and suggest that it may be indicative of nuclear versus cytoplasmic staining, but that a method for validation would be necessary to more strongly support this claim (lines 629-634). Nonetheless, we believe the temporal pattern of staining is consistent with the interpretation of puncta as nuclear and diffuse as cytoplasmic staining (lines 629-634). "4. The authors describe the euthanasia protocol by placing animals for 5 min in ice-cold water. Could this treatment not lead to a decrease in c-fos expression levels? A comment about this potential problem, or why the authors may not consider this to be problematic, should be added to the methods part." We do not anticipate that this is an issue, although we note in our bench protocol that we now recommend placing animals in ice water for 1 minute to minimize any degradation. The exact timing here may be subject to approval by local IACUC committees. We do not think this is an issue with respect to our data because (1) we can clearly see a difference in the amount of staining at different time points (Figure 4C) and (2) we can even capture differences in patterns of staining (puncta versus diffuse; Figure 4D). If we were getting large-scale degradation, it is unlikely we would be able to detect such subtle differences. Finally, we did a brief experiment in the lab and found that there was no difference in the number of cells detected if animals are left in ice for 1 minute versus 5 minutes (mentioned in line 151-152). "5. The authors should discuss the time course experiments of c-fos expression more rigorously. It is already quite detailed and good, but the fact that c-fos expression decays again after 15 minutes does not have to imply that c-fos can not accumulate even more - it may also simply mean that fish get habituated to the novel tank, and neuronal activity in the areas starts to decay again." We believe this comment may have arisen due to a misunderstanding of our experimental manipulation. In our paradigm, fish are placed in the novel tank for 6 minutes and then placed back in their home tank for variable periods of time (5, 15, 30, 60, or 120 minutes). Thus, all fish for all time points were exposed to the novel tank for the same amount of time. In this way, there wouldn't be any potential for habituation to the novel tank to contribute to our cfos results. Nonetheless, we appreciate the comment because it suggests that our manuscript was not as clear as we would have liked and prompted us to make our design clearer by adding an additional image (Figure 4A).

Re: methods "1. The authors say that they modified the iDisco protocol from the original version to be compatible with HCR FISH. Please explain briefly what those modifications are and why they think it mattered. In a similar vein, the authors may also discuss why they chose iDisco clearing and not one of the other existing methods." We optimized the iDISCO protocol to accommodate HCR labeling, making specific adjustments to enhance cfos signal brightness while minimizing background noise and reducing waste. We have now noted these changes on lines 171-173. There were other small changes to the washes etc. to make iDISCO and HCR compatible.

We have added why we chose iDISCO to lines 783-788 of the discussion. In brief, it was because the adult zebrafish brain atlas was made using iDISCO, so we wanted to ensure our images looked as similar as possible to the images in AZBA for registration, and prior work has found that iDISCO is compatible with in situ HCR. "2. Due to its toxicity, iDisco requires the usage of specialized enclosed chambers and optics, making this clearing method quite complex for many labs. The authors only briefly say: "Samples were mounted on the sample holder using an ultraviolet cured resin (NOA 61, Norland Products, Jamesburg, NJ) with a refractive index (1.56) that matched DBE." It would be nice to expand on this, helping readers to decide whether iDisco clearing and HCR FISH would indeed be a useful approach in their labs. Can the authors add an image of the sample holder inside the light sheet microscope to the supplement?" We chose iDISCO because it yields robust, consistent clearing. It is also the technique used in the making of the adult zebrafish brain atlas (AZBA), ensuring our autofluorescence images would be as similar as possible, which is important for registration. We appreciate that not every lab will have access to a light-sheet microscope compatible with iDISCO. However, some, like the MIltenyi Ultramicroscope II, were designed specifically to be compatible with imaging in organic solvents. We have now added this as a limitation to our study (lines 783-794).

We have also added an image of the sample holder and how brains are mounted (Extended Figure 2-1). "3. How do the authors exclude that the clearing treatment itself induces the diversity of mRNA distributions (nuclear vs. cytosolic)? Can the authors provide a dataset of a non-cleared fish from their light-sheet system or from the published literature that shows nuclear-localized mRNA as well? In Figure 2d, how do they know that the c-fos is clustered inside the nucleus, and not just somewhere at a specific location in the cytosol? Do they have an indication of the location of the cell body from the auto-fluorescent images or a DAPI staining?" We do not believe our treatment is resulting in the diversity of mRNA distributions. If this were the case, we would not expect to see the shift in distribution based on the time after exposure to the novel tank (Figure 4D) where the number of cells containing puncta increases faster than those with diffuse staining. We believe the most parsimonious explanation is that we are capturing the initial burst of cfos transcription in the nucleus (at 5 min) that then shifts to the cytoplasm (at 15 min). This is consistent with the time it is thought to take for mRNA to be transcribed and shuttled from the nucleus to the cytoplasm (Oeffinger and Zenklusen, 2012). We now include a discussion of these points in the paper (lines 629-634). As mentioned above, we were not able to include a nuclear stain for technical reasons and the autofluorescence image does not give any indication of where the nucleus is. Nonetheless, we think the data is highly suggestive that the puncta represent nuclear staining whereas the diffuse staining is cytoplasmic. But we note that this is speculative at this point (lines 633-634). "4. The use of autofluorescence as a means for registration is potentially interesting yet needs some more evidence to be convincing. The authors even mention challenges with this approach. Showing examples of alignment of the autofluorescence channel, reliable alignment of some neuroanatomical region, and providing some quantification for efficacy would be a significant improvement, especially as this is one of the novel elements of the manuscript.

We thank the reviewers for this comment as it pushed us to generate more convincing data showing the quality of our segmentation. We have now added this to Figure 3 (Figure 3C and D). We did perform quantification of image similarity between AZBA and images in the present study across the different parameters that we tested (Extended Figure 2-2) but these did not do a good job differentiating parameter sets. Indeed, to our knowledge, there are no agreed upon parameters that indicate 'good' segmentation. Thus, in the absence of clear quantitative measures, we have provided additional image segmentations as qualitative measures.

Minor issues "1. Line 117. What is the rationale for removing cell candidates within 9um of each other and what impact could this have on analysis/interpretation." We did this because the CellFinder algorithm tended to label one cell multiple times during the cell detection stage. If we modified the detection parameters to reduce this over labeling, we lost labelling in other parts of the brain. Our solution was to allow the overlabelling by the detection algorithm, but then to remove cells that were in close proximity after the fact. 9um was chosen empirically, it was a distance we found that removed overlapping cells without losing distinct cells in close proximity. We've now added more explanation of this in the methods section (lines 225-230). We do not believe this would have any appreciable impact on the interpretation of our data. "2. Figure 4. The use of an ANOVA for euthanasia time &sex needs further explanation. Were sex differences considered and/or relevant? Was the sex proportions kept consistent across groups?" We collected data from fish of both sexes (males and females; added to line 481 to clarify). We found that there was no significant effect of sex on the levels of cfos, only a 'trend' at P = 0.07, and only of a small effect size ( 2 = 0.059). We added a line to the results indicating that this trend was in the direction of females having slightly higher levels of cfos than males (lines 490-492). The proportions of animals of each sex were approximately equal across groups (n's are in the caption for Figure 4). "3. In various parts of the manuscript the use or testing of various species is mentioned yet may be overstated without additional testing as overall brain size, lipid composition, and numerous other factors may not allow the zebrafish techniques to be transferred. This may be worth limiting to a discussion point." We have now removed references to the fact that our approach may be applicable to other species. "4. Specifically mentioning the amgydala in the abstract is somewhat distracting as it never becomes a significant point again." Mention of the subpallial amygdala is limited to the significance statement. It is mentioned again in the discussion, as some parts of the ventral telencephalon are thought to be homologous to the mammalian subpallial amygdala (lines 701-703). "5. Line 69. Does 'this behavioral repertoire' refer to adult behavior?" Yes, this refers to adults. We have modified it so it reads "this adult behavioral repertoire" (line 70) to make this clearer. "6. In the methods it may be worth combining HCR labeling and clearing. As is the timing and combination of these two techniques is unclear and would seem to be important for another group to perform these techniques." We have now combined these to make it clearer that this is a combined method. "7. Line 488 sheet numerical aperture of XX?" We apologize for not filling this in. It has now been updated to be a sheet aperture of 0.156 (line 206). "8. It would be nice to add information about which statistical test was used in Figure 4 (as described in the methods, this should be a Dunnet's t-test). What do the confidence intervals indicate in Figure 4? (std?). Please add this information to the legend. There is only one star (0 vs. 15 min). Does this mean all other tests were not significant? Please add a note in the legend about this point." This information has now been added to the figure caption for figure 4. The error bars are standard error of the mean. And only significant comparisons are indicated. All other comparisons were not significant. We've now also added this point to the results (lines 493-494) "9. Probably it's the review version of the document, but in the final paper, a higher resolution version of Figure 2 would be good." We have made sure high-resolution images are uploaded for the second review. "10. The usage of two color maps in Figure 6 is confusing. Could the authors label the brain region name initials in a color based on the super-region, on both the x and y axis? Or use thin colored lines on both axes? This may be a better way to allow one to identify which brain region is (anti)correlated with which brain region." We have now altered Figure 6 so that the color map containing the higher order regions overlaps with the individual region names. We thank the reviewers for this comment as it has increased the readability of this figure.

We would also like to note that during our additional analysis we noticed that the olfactory bulbs for two of the brains for the 15-minute group did not register properly (noted in lines 354-355), and so we removed the counts from these two brains from the analysis. This is likely due to the fact that this region tends to be somewhat variable between animals and has a bit more tendency to be stretched or altered during dissection. We have updated the functional connectome analysis (lines 537-581). This change resulted in the olfactory bulbs as being identified as important for the network. We have updated the results in include this point (lines 553-559), updated the abstract, and added a paragraph to the discussion (lines 841-758).

Thank you for considering our manuscript for publication.

Sincerely, The authors

## References

[B1] Ahrens MB, Li JM, Orger MB, Robson DN, Schier AF, Engert F, Portugues R (2012) Brain-wide neuronal dynamics during motor adaptation in zebrafish. Nature 485:471–477. 10.1038/nature11057 22622571 PMC3618960

[B2] Akinrinade I, Kareklas K, Teles MC, Reis TK, Gliksberg M, Petri G, Levkowitz G, Oliveira RF (2023) Evolutionarily conserved role of oxytocin in social fear contagion in zebrafish. Science 379:1232–1237. 10.1126/science.abq5158 36952426

[B3] Avants B, Tustison NJ, Song G (2009) Advanced normalization tools: v1.0. Insight J 2:1–35. 10.54294/uvnhin

[B4] Avants BB, Tustison NJ, Song G, Cook PA, Klein A, Gee JC (2011) A reproducible evaluation of ANTs similarity metric performance in brain image registration. Neuroimage 54:2033–2044. 10.1016/j.neuroimage.2010.09.025 20851191 PMC3065962

[B5] Baraban SC, Taylor MR, Castro PA, Baier H (2005) Pentylenetetrazole induced changes in zebrafish behavior, neural activity and c-fos expression. Neuroscience 131:759–768. 10.1016/j.neuroscience.2004.11.031 15730879

[B6] Beigloo F, Davidson CJ, Gjonaj J, Perrine SA, Kenney JW (2024) Individual differences in the boldness of female zebrafish are associated with alterations in serotonin function. J Exp Biol 227:jeb247483. 10.1242/jeb.247483 38842023 PMC11213521

[B7] Ben-Ami Bartal I, et al. (2021) Neural correlates of ingroup bias for prosociality in rats. Elife 10:e65582. 10.7554/eLife.65582 34253289 PMC8277352

[B8] Benjamini Y, Hochberg Y (1995) Controlling the false discovery rate: a practical and powerful approach to multiple testing. J R Stat Soc Series B Methodol 57:289–300. 10.1111/j.2517-6161.1995.tb02031.x

[B9] Bisong E (2019) Google colaboratory. In: *Building machine learning and deep learning models on google cloud platform: a comprehensive guide for beginners* (Bisong E, ed), pp 59–64. Berkeley CA: Apress. 10.1007/978-1-4842-4470-8_7

[B10] Blaser RE, Chadwick L, McGinnis GC (2010) Behavioral measures of anxiety in zebrafish (*Danio rerio*). Behav Brain Res 208:56–62. 10.1016/j.bbr.2009.11.009 19896505

[B11] Blondel VD, Guillaume J-L, Lambiotte R, Lefebvre E (2008) Fast unfolding of communities in large networks. J Stat Mech Theory Exp 2008:P10008. 10.1088/1742-5468/2008/10/P10008

[B12] Bria A, Iannello G (2012) TeraStitcher - a tool for fast automatic 3D-stitching of teravoxel-sized microscopy images. BMC Bioinformatics 13:316. 10.1186/1471-2105-13-316 23181553 PMC3582611

[B13] Brockerhoff SE, Hurley JB, Janssen-Bienhold U, Neuhauss SC, Driever W, Dowling JE (1995) A behavioral screen for isolating zebrafish mutants with visual system defects. Proc Natl Acad Sci U S A 92:10545–10549. 10.1073/pnas.92.23.10545 7479837 PMC40648

[B14] Bullmore ET, Bassett DS (2011) Brain graphs: graphical models of the human brain connectome. 10.1146/annurev-clinpsy-040510-143934

[B15] Bullmore E, Sporns O (2009) Complex brain networks: graph theoretical analysis of structural and functional systems. Nat Rev Neurosci 10:186–198. 10.1038/nrn2575 19190637

[B16] Bundschuh ST, Zhu P, Schärer Y-PZ, Friedrich RW (2012) Dopaminergic modulation of mitral cells and odor responses in the zebrafish olfactory bulb. J Neurosci 32:6830–6840. 10.1523/JNEUROSCI.6026-11.2012 22593052 PMC6622199

[B17] Burgess HA, Burton EA (2023) A critical review of zebrafish neurological disease models−1. the premise: neuroanatomical, cellular and genetic homology and experimental tractability. Oxf Open Neurosci 2:kvac018. 10.1093/oons/kvac018 37649777 PMC10464506

[B18] Butler JM, Whitlow SM, Roberts DA, Maruska KP (2018) Neural and behavioural correlates of repeated social defeat. Sci Rep 8:6818. 10.1038/s41598-018-25160-x 29717159 PMC5931592

[B19] Byrd CA, Brunjes PC (1995) Organization of the olfactory system in the adult zebrafish: histological, immunohistochemical, and quantitative analysis. J Comp Neurol 358:247–259. 10.1002/cne.903580207 7560285

[B20] Cachat J, et al. (2010) Measuring behavioral and endocrine responses to novelty stress in adult zebrafish. Nat Protoc 5:1786–1799. 10.1038/nprot.2010.140 21030954

[B21] Cargnello M, Roux PP (2011) Activation and function of the MAPKs and their substrates, the MAPK-activated protein kinases. Microbiol Mol Biol Rev 75:50–83. 10.1128/mmbr.00031-10 21372320 PMC3063353

[B22] Choi HMT, Schwarzkopf M, Fornace ME, Acharya A, Artavanis G, Stegmaier J, Cunha A, Pierce NA (2018) Third-generation in situ hybridization chain reaction: multiplexed, quantitative, sensitive, versatile, robust. Development 145:dev165753. 10.1242/dev.165753 29945988 PMC6031405

[B23] Chung L (2015) A brief Introduction to the transduction of neural activity into Fos signal. Dev Reprod 9:61–67. 10.12717/DR.2015.19.2.061 27004262 PMC4801051

[B24] Claudi F, Petrucco L, Tyson A, Branco T, Margrie T, Portugues R (2020) BrainGlobe Atlas API: a common interface for neuroanatomical atlases. J Open Source Softw 5:2668. 10.21105/joss.02668

[B25] Clayden J, Cox B, Jenkinson M, Reynolds R, Fissell K, Gailly J, Adler M (2021) RNifti: Fast R and C++ access to NIfTI images. R Package Version 170:1. 10.32614/CRAN.package.RNifti

[B26] Clemente D, Porteros Á, Weruaga E, Alonso JR, Arenzana FJ, Aijón J, Arévalo R (2004) Cholinergic elements in the zebrafish central nervous system: histochemical and immunohistochemical analysis. J Comp Neurol 474:75–107. 10.1002/cne.20111 15156580

[B27] Corradi L, Bruzzone M, Maschio dal M, Sawamiphak S, Filosa A (2022) Hypothalamic galanin-producing neurons regulate stress in zebrafish through a peptidergic, self-inhibitory loop. Curr Biol 32:1497–1510.e5. 10.1016/j.cub.2022.02.011 35219430

[B28] Croy I, Hummel T (2016) Olfaction as a marker for depression. J Neurol 264:631–638. 10.1007/s00415-016-8227-8 27393116

[B29] Csardi G, Nepusz T (2006) The igraph software package for complex network research. InterJ Complex Syst 1695:1–9. 10.5281/zenodo.7682609

[B30] Ding JM, Carver WC, Terracio L, Buggy J (1994) Proto-oncogene c-*fos* and the regulation of vasopressin gene expression during dehydration. Mol Brain Res 21:247–255. 10.1016/0169-328X(94)90255-0 8170349

[B31] Dodt HU, Saghafi S, Becker K, Jährling N, Niendorf A, Hahn C, Pende M, Wanis M (2015) Ultramicroscopy: development and outlook. Neurophotonics 2:041407. 10.1117/1.NPh.2.4.041407 26730396 PMC4696521

[B32] Dunn TW, Mu Y, Narayan S, Randlett O, Naumann EA, Yang C-T, Schier AF, Freeman J, Engert F, Ahrens MB (2016) Brain-wide mapping of neural activity controlling zebrafish exploratory locomotion. Elife 5:e12741. 10.7554/eLife.12741 27003593 PMC4841782

[B33] Edwards JG, Greig A, Sakata Y, Elkin D, Michel WC (2007) Cholinergic innervation of the zebrafish olfactory bulb. J Comp Neurol 504:631–645. 10.1002/cne.21480 17722029

[B34] Erdös P, Rényi A (1960) On the evolution of random graphs. Publ Math Inst Hung Acad Sci 5:17–60.

[B35] Fadok JP, Markovic M, Tovote P, Lüthi A (2018) New perspectives on central amygdala function. Curr Opin Neurobiol 49:141–147. 10.1016/j.conb.2018.02.009 29522976

[B36] Fallani FDV, Latora V, Chavez M (2017) A topological criterion for filtering information in complex brain networks. PLoS Comput Biol 13:e1005305. 10.1371/journal.pcbi.1005305 28076353 PMC5268647

[B37] Gerlai R (2014) Social behavior of zebrafish: from synthetic images to biological mechanisms of shoaling. J Neurosci Methods 234:59–65. 10.1016/j.jneumeth.2014.04.028 24793400

[B38] Gerlai R (2020) Evolutionary conservation, translational relevance and cognitive function: the future of zebrafish in behavioral neuroscience. Neurosci Biobehav Rev 116:426–435. 10.1016/j.neubiorev.2020.07.009 32681940

[B39] Gerlai R (2023) Zebrafish (*Danio rerio*): a newcomer with great promise in behavioral neuroscience. Neurosci Biobehav Rev 144:104978. 10.1016/j.neubiorev.2022.104978 36442644

[B40] Gholipour A, Kehtarnavaz N, Briggs R, Devous M, Gopinath K (2007) Brain functional localization: a survey of image registration techniques. IEEE Trans Med Imaging 26:427–451. 10.1109/TMI.2007.892508 17427731

[B41] Grillner S, Robertson B (2016) The basal ganglia over 500 million years. Curr Biol 26:R1088–R1100. 10.1016/j.cub.2016.06.041 27780050

[B42] Grunwald DJ, Kimmel CB, Westerfield M, Walker C, Streisinger G (1988) A neural degeneration mutation that spares primary neurons in the zebrafish. Dev BIiol 126:115–128. 10.1016/0012-1606(88)90245-X 3342929

[B43] Guimerà R, Amaral LAN (2005) Cartography of complex networks: modules and universal roles. J Stat Mech 2005:1–13. 10.1088/1742-5468/2005/02/P02001 18159217 PMC2151742

[B44] Guzowski JF, Setlow B, Wagner EK, McGaugh JL (2001) Experience-dependent gene expression in the rat hippocampus after spatial learning: a comparison of the immediate-early GenesArc, c-fos, and zif268. J Neurosci 21:5089–5098. 10.1523/JNEUROSCI.21-14-05089.2001 11438584 PMC6762831

[B45] Herget U, Wolf A, Wullimann MF, Ryu S (2014) Molecular neuroanatomy and chemoarchitecture of the neurosecretory preoptic-hypothalamic area in zebrafish larvae. J Comp Neurol 522:1542–1564. 10.1002/cne.23480 24127437

[B46] Hillman EMC, Voleti V, Li W, Yu H (2019) Light-sheet microscopy in neuroscience. Annu Rev Neurosci 42:295–313. 10.1146/annurev-neuro-070918-050357 31283896 PMC6800245

[B48] Humphries MD, Gurney K (2008) Network ‘small-world-ness’: a quantitative method for determining canonical network equivalence. PLoS One 3:e0002051. 10.1371/journal.pone.0002051 18446219 PMC2323569

[B49] Jones LJ, Norton WHJ (2015) Using zebrafish to uncover the genetic and neural basis of aggression, a frequent comorbid symptom of psychiatric disorders. Behav Brain Res 276:171–180. 10.1016/j.bbr.2014.05.055 24954772

[B50] Kacprzak V, Patel NA, Riley E, Yu L, Yeh J-RJ, Zhdanova IV (2017) Dopaminergic control of anxiety in young and aged zebrafish. Pharmacol Biochem Behav 157:1–8. 10.1016/j.pbb.2017.01.005 28408289 PMC5505502

[B51] Kalueff AV, et al. (2013) Towards a comprehensive catalog of zebrafish behavior 1.0 and beyond. Zebrafish 10:70–86. 10.1089/zeb.2012.0861 23590400 PMC3629777

[B52] Kareklas K, Teles MC, Nunes AR, Oliveira RF (2023) Social zebrafish: *Danio rerio* as an emerging model in social neuroendocrinology. J Neuroendocrinol 35:e13280. 10.1111/jne.13280 37165563

[B53] Kawai T, Abe H, Oka Y (2012) Dopaminergic neuromodulation of synaptic transmission between mitral and granule cells in the teleost olfactory bulb. J Neurophysiol 107:1313–1324. 10.1152/jn.00536.2011 22157125

[B54] Kenney JW (2020) Associative and nonassociative learning in adult zebrafish. In: *Behavioral and neural genetics of zebrafish* (Gerlai RT, ed), pp 187–204, San Diego: Elsevier. 10.1016/b978-0-12-817528-6.00012-7

[B55] Kenney JW, Sorokina O, Genheden M, Sorokin A, Armstrong JD, Proud CG (2015) Dynamics of elongation factor 2 kinase regulation in cortical neurons in response to synaptic activity. J Neurosci 35:3034–3047. 10.1523/JNEUROSCI.2866-14.2015 25698741 PMC4331626

[B56] Kenney JW, Steadman PE, Young O, Shi MT, Polanco M, Dubaishi S, Covert K, Mueller T, Frankland PW (2021) A 3D adult zebrafish brain atlas (AZBA) for the digital age. Elife 10:e69988. 10.7554/eLife.69988 34806976 PMC8639146

[B57] Kermen F, Lal P, Faturos NG, Yaksi E (2020) Interhemispheric connections between olfactory bulbs improve odor detection. PLoS Biol 18:e3000701. 10.1371/journal.pbio.3000701 32310946 PMC7192517

[B58] Klein A, et al. (2009) Evaluation of 14 nonlinear deformation algorithms applied to human brain MRI registration. Neuroimage 46:786–802. 10.1016/j.neuroimage.2008.12.037 19195496 PMC2747506

[B59] Kovács KJ (1998) Invited review c-Fos as a transcription factor: a stressful (re)view from a functional map. Neurochem Int 33:287–297. 10.1016/S0197-0186(98)00023-0 9840219

[B60] Kramer EE, Steadman PE, Epp JR, Frankland PW, Josselyn SA (2018) Assessing individual neuronal activity across the intact brain: using hybridization chain reaction (HCR) to detect *Arc* mRNA localized to the nucleus in volumes of cleared brain tissue. Curr Protoc Neurosci 84:e49. 10.1002/cpns.49 29944213

[B61] Kumar V, Krolewski DM, Hebda-Bauer EK, Parsegian A, Martin B, Foltz M, Akil H, Watson SJ (2021) Optimization and evaluation of fluorescence in situ hybridization chain reaction in cleared fresh-frozen brain tissues. Brain Struct Funct 226:481–499. 10.1007/s00429-020-02194-4 33386994 PMC7962668

[B62] Lau BYB, Mathur P, Gould GG, Guo S (2011) Identification of a brain center whose activity discriminates a choice behavior in zebrafish. Proc Natl Acad Sci U S A 108:2581–2586. 10.1073/pnas.1018275108 21262817 PMC3038752

[B63] Leonard BE, Tuite M (1981) Anatomical, physiological, and behavioral aspects of olfactory bulbectomy in the rat. Int Rev Neurobiol 22:251–286. 10.1016/S0074-7742(08)60295-0 7024168

[B64] Loring MD, Thomson EE, Naumann EA (2020) Whole-brain interactions underlying zebrafish behavior. Curr Opin Neurobiol 65:88–99. 10.1016/j.conb.2020.09.011 33221591 PMC10697041

[B65] Luca RM, Gerlai R (2012) In search of optimal fear inducing stimuli: differential behavioral responses to computer animated images in zebrafish. Behav Brain Res 226:66–76. 10.1016/j.bbr.2011.09.001 21920389 PMC3203217

[B66] Lucibello FC, Lowag C, Neuberg M, Müller R (1989). Trans-repression of the mouse c-fos promoter: a novel mechanism of Fos-mediated trans-regulation. Cell 59:999–1007. 10.1016/0092-8674(89)90756-3 2513130

[B67] Marquart GD, Tabor KM, Horstick EJ, Brown M, Geoca AK, Polys NF, Nogare DD, Burgess HA (2017) High-precision registration between zebrafish brain atlases using symmetric diffeomorphic normalization. Gigascience 6:gix056. 10.1093/gigascience/gix056 28873968 PMC5597853

[B68] Maruska KP, Butler JM, Field KE, Forester C, Augustus A (2020) Neural activation patterns associated with maternal mouthbrooding and energetic state in an African cichlid fish. Neuroscience 446:199–212. 10.1016/j.neuroscience.2020.07.025 32707292

[B69] Maximino C, Puty B, Benzecry R, Araújo J, Lima MG, de Jesus Oliveira Batista E, Renata de Matos Oliveira K, Crespo-Lopez ME, Herculano AM (2013) Role of serotonin in zebrafish (*Danio rerio*) anxiety: relationship with serotonin levels and effect of buspirone, WAY 100635, SB 224289, fluoxetine and para-chlorophenylalanine (pCPA) in two behavioral models. Neuropharmacology 71:83–97. 10.1016/j.neuropharm.2013.03.006 23541719

[B70] McMillan SC, Géraudie J, Akimenko M-A (2015) Pectoral fin breeding tubercle clusters: a method to determine zebrafish sex. Zebrafish 12:121–123. 25548979 10.1089/zeb.2014.1060 PMC4298160

[B71] Miyasaka N, Arganda-Carreras I, Wakisaka N, Masuda M, Sümbül U, Seung HS, Yoshihara Y (2014) Olfactory projectome in the zebrafish forebrain revealed by genetic single-neuron labelling. Nat Commun 5:3639. 10.1038/ncomms4639 24714622

[B72] Miyasaka N, Morimoto K, Tsubokawa T, Higashijima S, Okamoto H, Yoshihara Y (2009) From the olfactory bulb to higher brain centers: genetic visualization of secondary olfactory pathways in zebrafish. J Neurosci 29:4756–4767. 10.1523/JNEUROSCI.0118-09.2009 19369545 PMC6665349

[B73] Mueller T (2022) The everted amygdala of ray-finned fish: zebrafish makes a case. Brain Behav Evol 97:321–335. 10.1159/000525669 35760049

[B74] Murphy K, et al. (2011) Evaluation of registration methods on thoracic CT: the EMPIRE10 challenge. IEEE Trans Med Imaging 30:1901–1920. 10.1109/TMI.2011.2158349 21632295

[B75] Nabinger DD, Altenhofen S, Buatois A, Facciol A, Peixoto JV, da Silva JMK, Chatterjee D, Rübensam G, Gerlai R, Bonan CD (2023) Acute administration of a dopamine D2/D3 receptor agonist alters behavioral and neural parameters in adult zebrafish. Prog Neuropsychopharmacol Biol Psychiatry 125:110753. 10.1016/j.pnpbp.2023.110753 36934998

[B76] Nowicki M, Tran S, Muraleetharan A, Markovic S, Gerlai R (2014) Serotonin antagonists induce anxiolytic and anxiogenic-like behavior in zebrafish in a receptor-subtype dependent manner. Pharmacol Biochem Behav 126:170–180. 10.1016/j.pbb.2014.09.022 25284132

[B77] Oeffinger M, Zenklusen D (2012) To the pore and through the pore: a story of mRNA export kinetics. Biochim Biophys Acta 1819:494–506. 10.1016/j.bbagrm.2012.02.011 22387213 PMC3345096

[B78] Palieri V, Paoli E, Wu YK, Haesemeyer M, Kadow ICG, Portugues R (2024) The preoptic area and dorsal habenula jointly support homeostatic navigation in larval zebrafish. Curr Biol 34:489–504.e7. 10.1016/j.cub.2023.12.030 38211586 PMC10849091

[B79] Parada CDC, Mayer U, Chagnaud BP (2024) The dorsal part of the anterior tuberal nucleus responds to auditory stimulation in zebrafish (*Danio rerio*). eNeuro 11:ENEURO.0062-24.2024. 10.1523/ENEURO.0062-24.2024 38918052 PMC11236576

[B80] Pinho JS, Cunliffe V, Kareklas K, Petri G, Oliveira RF (2023) Social and asocial learning in zebrafish are encoded by a shared brain network that is differentially modulated by local activation. Commun Biol 6:1–13. 10.1038/s42003-023-04999-5 37308619 PMC10260970

[B81] Porter BA, Mueller T (2020) The zebrafish amygdaloid complex – functional ground plan, molecular delineation, and everted topology. Front Neurosci 14:608. 10.3389/fnins.2020.00608 32765204 PMC7378821

[B82] Portugues R, Feierstein CE, Engert F, Orger MB (2014) Whole-Brain activity maps reveal stereotyped, distributed networks for visuomotor behavior. Neuron 81:1328–1343. 10.1016/j.neuron.2014.01.019 24656252 PMC4448760

[B83] Raam T, Hong W (2021) Organization of neural circuits underlying social behavior: a consideration of the medial amygdala. Curr Opin Neurobiol 68:124–136. 10.1016/j.conb.2021.02.008 33940499 PMC8243811

[B84] Rajput N, Parikh K, Kenney JW (2022) Beyond bold versus shy: zebrafish exploratory behavior falls into several behavioral clusters and is influenced by strain and sex. Biol Open 11:bio059443. 10.1242/bio.059443 36039864 PMC9450886

[B85] Randlett O, et al. (2015) Whole-brain activity mapping onto a zebrafish brain atlas. Nat Methods 12:1039–1046. 10.1038/nmeth.3581 26778924 PMC4710481

[B47] R Core Team (2016) R: a language and environment for statistical computing. R Foundation Stat Comput 1:409. 10.1007/978-3-540-74686-7

[B86] Renier N, et al. (2016) Mapping of brain activity by automated volume analysis of immediate early genes. Cell 165:1789–1802. 10.1016/j.cell.2016.05.007 27238021 PMC4912438

[B87] Renier N, Wu Z, Simon DJ, Yang J, Ariel P, Tessier-Lavigne M (2014) IDISCO: a simple, rapid method to immunolabel large tissue samples for volume imaging. Cell 159:896–910. 10.1016/j.cell.2014.10.010 25417164

[B88] Reynaud EG, Peychl J, Huisken J, Tomancak P (2015) Guide to light-sheet microscopy for adventurous biologists. Nat Methods 12:30–34. 10.1038/nmeth.3222 25549268

[B89] Richardson DS, Guan W, Matsumoto K, Pan C, Chung K, Ertürk A, Ueda HR, Lichtman JW (2021) Tissue clearing. Nat Rev Methods Primers 1:1–24. 10.1038/s43586-021-00080-9 35128463 PMC8815095

[B90] Ruhl T, Zeymer M, von der Emde G (2017) Cannabinoid modulation of zebrafish fear learning and its functional analysis investigated by c-Fos expression. Pharmacol Biochem Behav 153:18–31. 10.1016/j.pbb.2016.12.005 27965084

[B91] Scaia MF, Akinrinade I, Petri G, Oliveira RF (2022) Sex differences in aggression are paralleled by differential activation of the brain social decision-making network in zebrafish. Front Behav Neurosci 16:784835. 10.3389/fnbeh.2022.784835 35250500 PMC8890505

[B92] Schweitzer J, Driever W (2009) Development of the dopamine systems in zebrafish development and engineering of dopamine neurons. 10.1007/978-1-4419-0322-8_1

[B93] Shainer I, Kuehn E, Laurell E, Al Kassar M, Mokayes N, Sherman S, Larsch J, Kunst M, Baier H (2023) A single-cell resolution gene expression atlas of the larval zebrafish brain. Sci Adv 9:eade9909. 10.1126/sciadv.ade9909 36812331 PMC9946346

[B94] Song C, Leonard BE (2005) The olfactory bulbectomised rat as a model of depression. Neurosci Biobehav Rev 29:627–647. 10.1016/j.neubiorev.2005.03.010 15925697

[B95] Spence R, Fatema MK, Reichard M, Huq KA, Wahab MA, Ahmed ZF, Smith C (2006) The distribution and habitat preferences of the zebrafish in Bangladesh. J Fish Biol 69:1435–1448. 10.1111/j.1095-8649.2006.01206.x

[B96] Toms CN, Echevarria DJ (2014) Back to basics: searching for a comprehensive framework for exploring individual differences in zebrafish (*Danio rerio*) behavior. Zebrafish 11:325–340. 10.1089/zeb.2013.0952 24921670

[B97] Tyson AL, Rousseau CV, Niedworok CJ, Keshavarzi S, Tsitoura C, Cossell L, Strom M, Margrie TW (2021) A deep learning algorithm for 3D cell detection in whole mouse brain image datasets. PLoS Comput Biol 17:e1009074. 10.1371/journal.pcbi.1009074 34048426 PMC8191998

[B98] Venincasa MJ, et al. (2021) Elevated preoptic brain activity in zebrafish glial glycine transporter mutants is linked to lethargy-like behaviors and delayed emergence from anesthesia. Sci Rep 11:3148. 10.1038/s41598-021-82342-w 33542258 PMC7862283

[B99] Verpeut JL, et al. (2023) Cerebellar contributions to a brainwide network for flexible behavior in mice. Commun Biol 6:1–17. 10.1038/s42003-023-04920-0 37277453 PMC10241932

[B100] Vetere G, Kenney JW, Tran LM, Xia F, Steadman PE, Parkinson J, Josselyn SA, Frankland PW (2017) Chemogenetic interrogation of a brain-wide fear memory network in mice. Neuron 94:363–374.e4. 10.1016/j.neuron.2017.03.037 28426969

[B101] Wheeler AL, Teixeira CM, Wang AH, Xiong X, Kovacevic N, Lerch JP, McIntosh AR, Parkinson J, Frankland PW (2013) Identification of a functional connectome for long-term fear memory in mice. PLoS Comput Biol 9:e1002853. 10.1371/journal.pcbi.1002853 23300432 PMC3536620

[B102] Wong K, et al. (2010) Analyzing habituation responses to novelty in zebrafish (*Danio rerio*). Behav Brain Res 208:450–457. 10.1016/j.bbr.2009.12.023 20035794

[B103] Wulliman MF, Rupp B, Reichert H (1996) Neuroanatomy of the zebrafish brain: a topological atlas. Basel: Birkhäuser Verlag.

[B104] Yushkevich PA, et al. (2019) User-Guided segmentation of multi-modality medical imaging datasets with ITK-SNAP. Neuroinformatics 17:83–102. 10.1007/s12021-018-9385-x 29946897 PMC6310114

[B105] Zangenehpour S, Chaudhuri A (2002) Differential induction and decay curves of *c-fos* and *zif268* revealed through dual activity maps. Mol Brain Res 109:221–225. 10.1016/S0169-328X(02)00556-9 12531532

