# Supplementary figures and images for: Whole-Brain Mapping in Adult Zebrafish and Identification of the Functional Brain Network Underlying the Novel Tank Test
Source: eNeuro. 2025 Mar 20;12(3):ENEURO.0382-24.2025. doi: 10.1523/ENEURO.0382-24.2025 (PMC11936448; doi:10.1523/ENEURO.0382-24.2025)

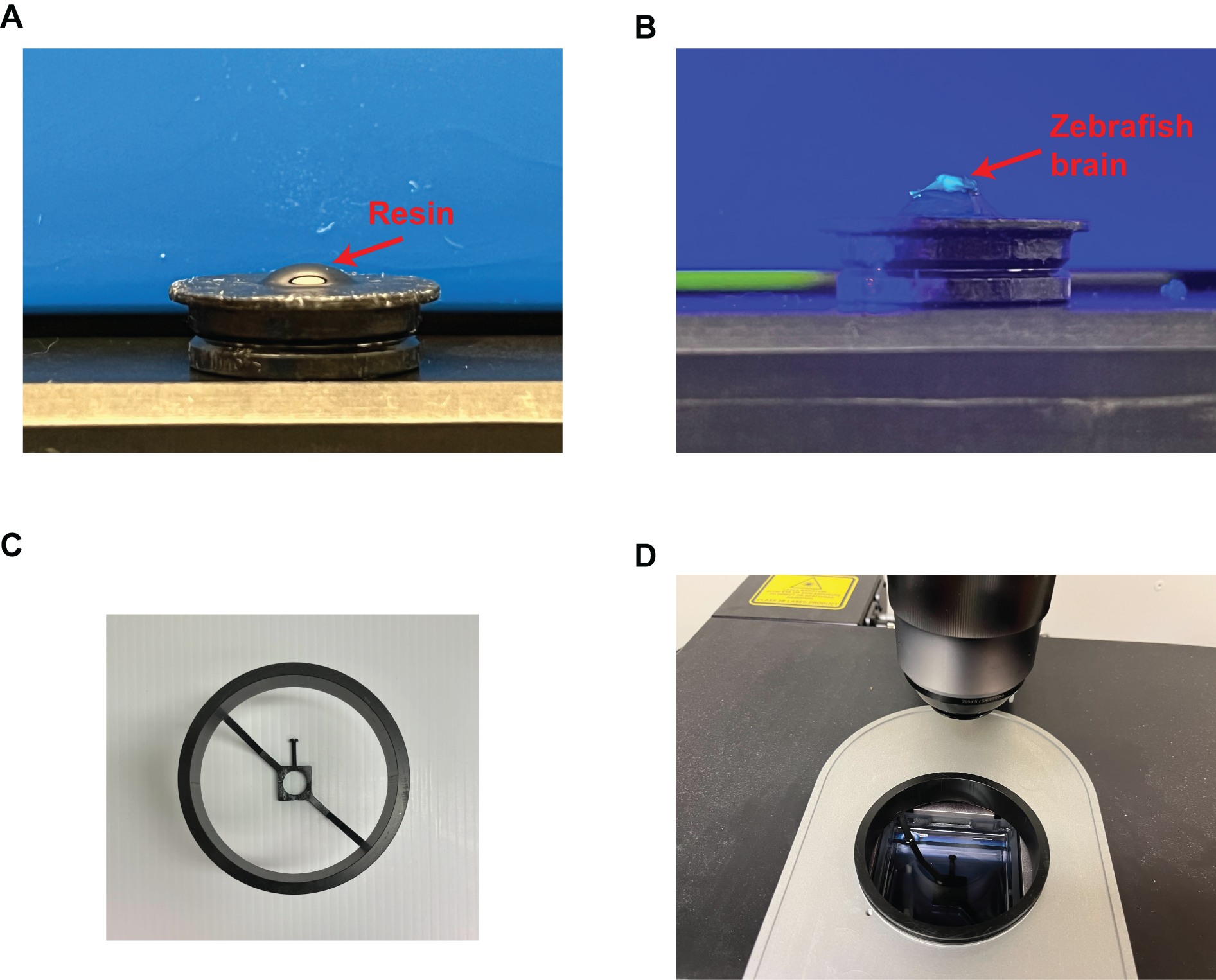

Supplement: Figure 2-1 — Mounting of the zebrafish brain for light sheet imaging. A) Ultraviolet cured resin is first used to form a hemisphere to raise the brain above the platform. B) Zebrafish brain mounted on top of the resin hemisphere. The brain is illuminated with ultraviolet light to make it visible. C) Sample holder where the platform is mounted before being placed in the imaging chamber. D) Sample holder in the imaging chamber of the Ultramicroscope II. Download Figure 2-1, TIF file. [file eneuro-12-ENEURO.0382-24.2025-s002.tif]

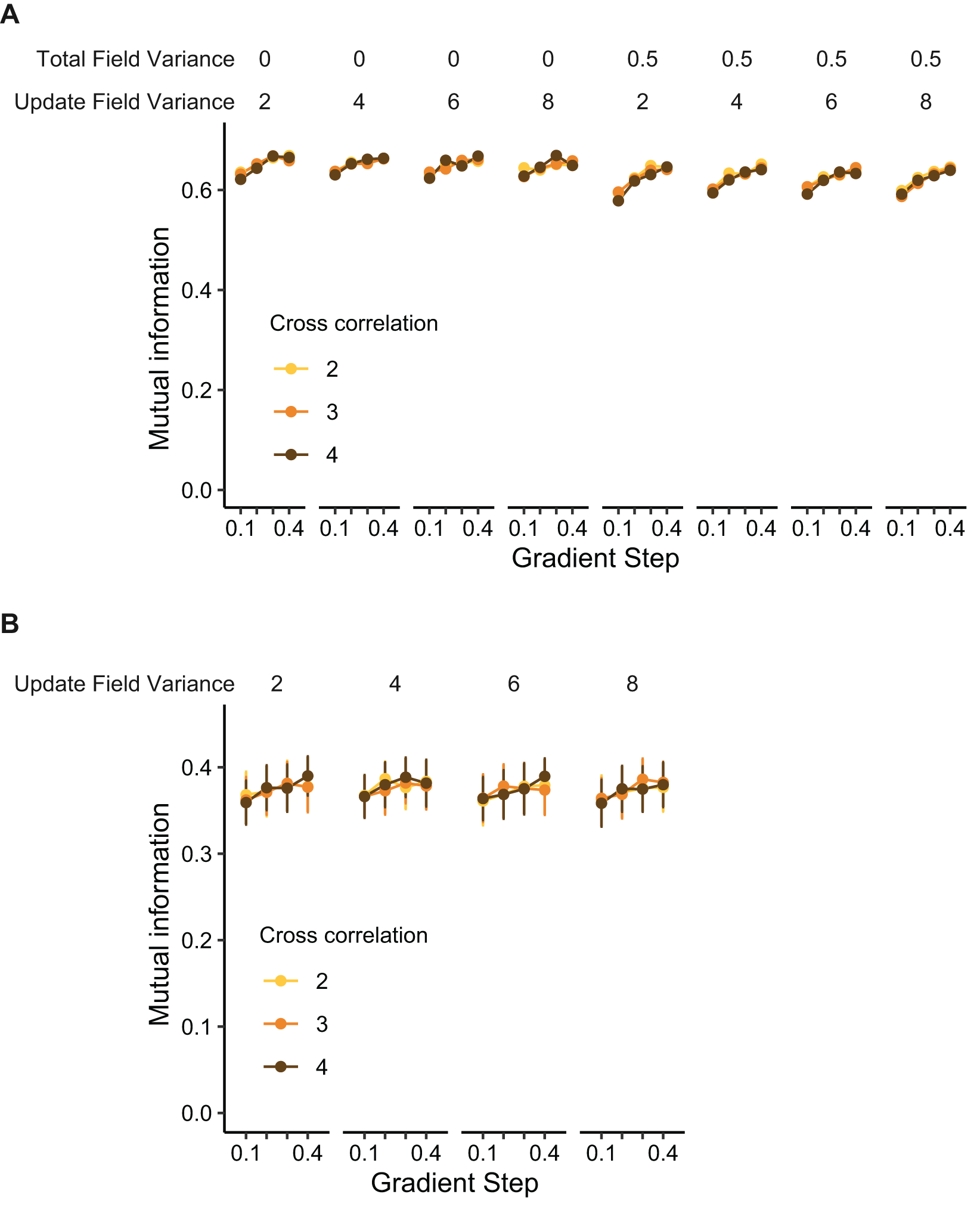

Supplement: Figure 2-2 — Download Figure 2-1, TIF file. [file eneuro-12-ENEURO.0382-24.2025-s009.tif]

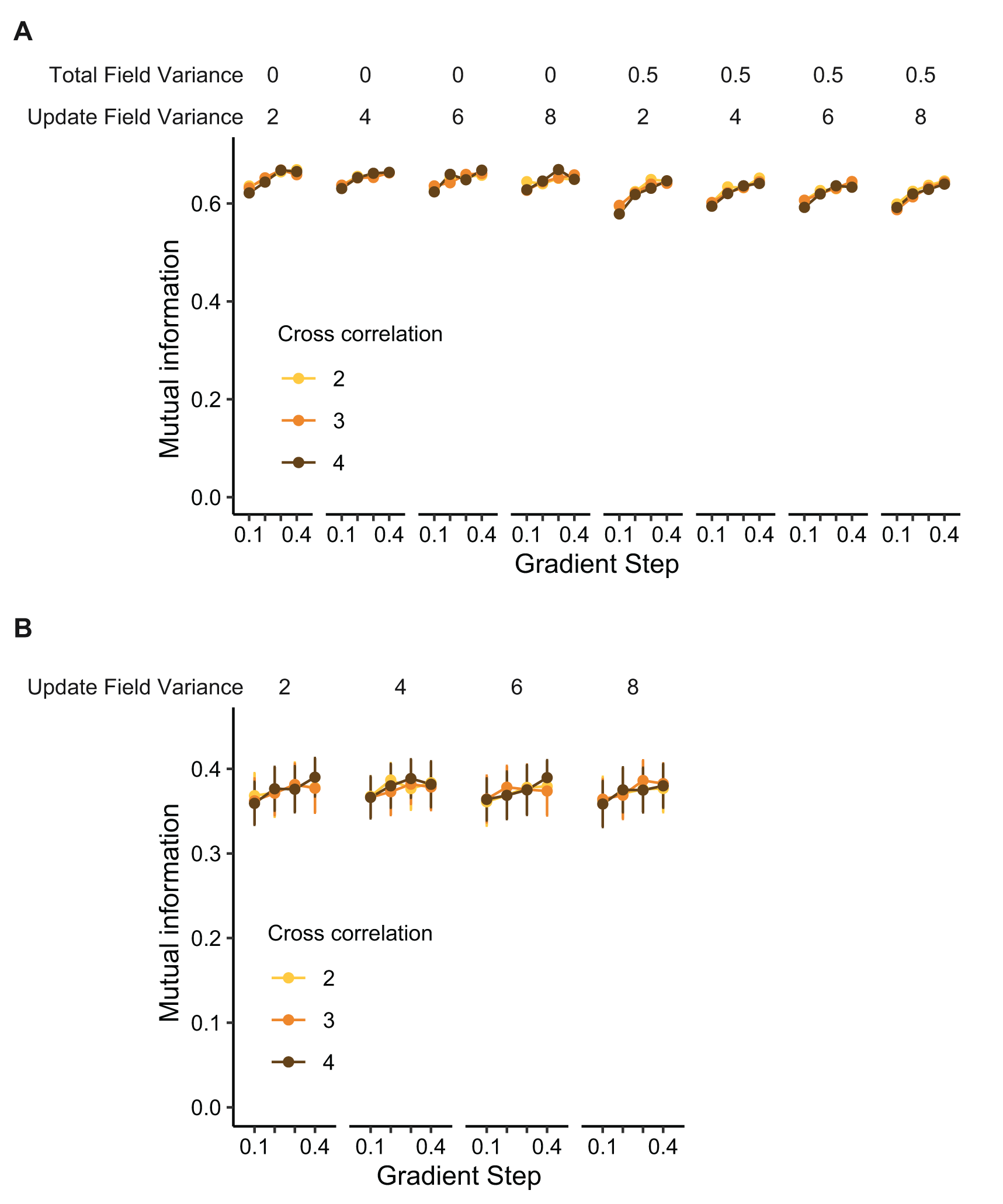

Supplement: Figure 3-1 — Quantitative assessment of image registration. A) Mutual information of the autofluorescence image from AZBA with the average template in the present study. B) Normalized mutual information of the autofluorescence image from AZBA registered to individual brains from the 15-minute cfos group. Error bars represent standard error of the mean; n=13. Download Figure 3-1, TIF file. [file eneuro-12-ENEURO.0382-24.2025-s003.tif]
