# Supplementary material for: Whole-Brain Mapping in Adult Zebrafish and Identification of the Functional Brain Network Underlying the Novel Tank Test
Source: eNeuro. 2025 Mar 20;12(3):ENEURO.0382-24.2025. doi: 10.1523/ENEURO.0382-24.2025 (PMC11936448; doi:10.1523/ENEURO.0382-24.2025)
Supplement: Table 4-1 — Architecture of CNN for detecting cytoplasmic and nuclear cfos stained cells. Download Table 4-1, DOCX file. [file eneuro-12-ENEURO.0382-24.2025-s004.docx]

Extended table 4-1. Architecture of CNN for detecting cytoplasmic and nuclear *cfos* stained cells.

| Layer (in order) | Output shape |
| --- | --- |
| Input layer | (None, 11, 11, 11, 1) |
| 3D convolution | (None, 11, 11, 11, 64) |
| ReLU | (None, 11, 11, 11, 64) |
| 3D max pooling | (None, 6, 6, 6, 64) |
| Batch normalization | (None, 6, 6, 6, 64) |
| 3D convolution | (None, 6, 6, 6, 64) |
| ReLu | (None, 6, 6, 6, 64) |
| 3D max pooling | (None, 3, 3, 3, 64) |
| Batch normalization | (None, 3, 3, 3, 64) |
| 3D convolution | (None, 3, 3, 3, 128) |
| ReLu | (None, 3, 3, 3, 128) |
| 3D max pooling | (None, 2, 2, 2, 128) |
| Batch normalization | (None, 2, 2, 2, 128) |
| 3D convolution | (None, 2, 2, 2, 256) |
| ReLu | (None, 2, 2, 2, 256) |
| 3D max pooling | (None, 1, 1, 1, 256) |
| Batch normalization | (None, 1, 1, 1, 256) |
| Global average pooling | (None, 256) |
| Dense | (None, 512) |
| ReLu | (None, 512) |
| Dropout | (None, 512) |
| Dense | (None, 1) |
| Activation | (None, 1) |
